# The emerging role of pyroptosis in pediatric cancers: from mechanism to therapy

**DOI:** 10.1186/s13045-022-01365-6

**Published:** 2022-10-08

**Authors:** Hua Wang, Xiaowen Zhou, Chenbei Li, Shuxiang Yan, Chengyao Feng, Jieyu He, Zhihong Li, Chao Tu

**Affiliations:** 1 Department of Orthopaedics, The Second Xiangya Hospital of Central South University, Changsha, 410011 Hunan China; 2 Hunan Key Laboratory of Tumor Models and Individualized Medicine, The Second Xiangya Hospital of Central South University, Changsha, 410011 Hunan China; 3 Xiangya School of Medicine, Central South University, Changsha, 410011 Hunan China; 4 Department of Geriatrics, The Second Xiangya Hospital of Central South University, Changsha, 410011 Hunan China

**Keywords:** Pyroptosis, Programmed cell death, Pediatric cancer, Osteosarcoma, Cytokine release syndrome

## Abstract

Pediatric cancers are the driving cause of death for children and adolescents. Due to safety requirements and considerations, treatment strategies and drugs for pediatric cancers have been so far scarcely studied. It is well known that tumor cells tend to progressively evade cell death pathways, which is known as apoptosis resistance, one of the hallmarks of cancer, dominating tumor drug resistance. Recently, treatments targeting nonapoptotic cell death have drawn great attention. Pyroptosis, a newly specialized form of cell death, acts as a critical physiological regulator in inflammatory reaction, cell development, tissue homeostasis and stress response. The action in different forms of pyroptosis is of great significance in the therapy of pediatric cancers. Pyroptosis could be induced and consequently modulate tumorigenesis, progression, and metastasis if treated with local or systemic therapies. However, excessive or uncontrolled cell death might lead to tissue damage, acute inflammation, or even cytokine release syndrome, which facilitates tumor progression or recurrence. Herein, we aimed to describe the molecular mechanisms of pyroptosis, to highlight and discuss the challenges and opportunities for activating pyroptosis pathways through various oncologic therapies in multiple pediatric neoplasms, including osteosarcoma, neuroblastoma, leukemia, lymphoma, and brain tumors.

## Introduction

**Fig. 1:**
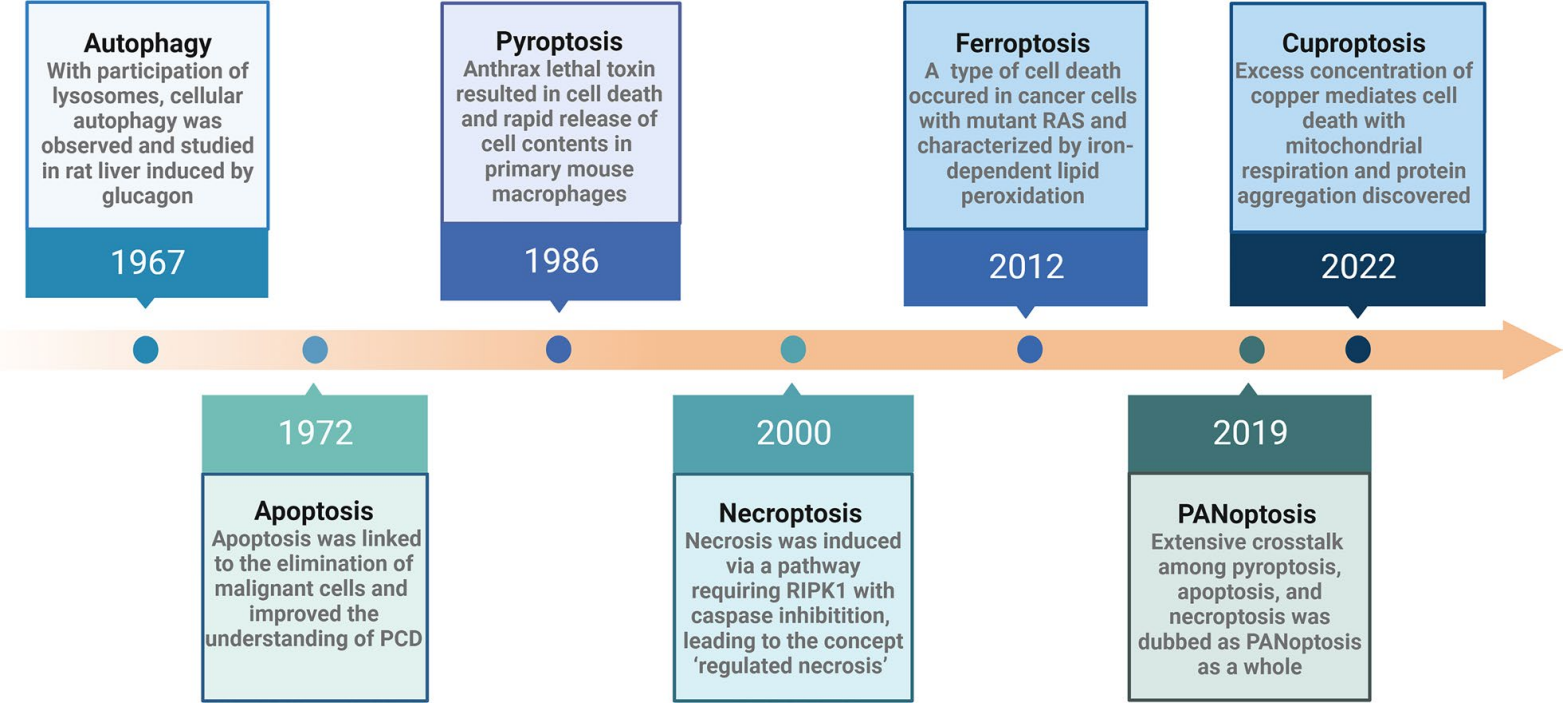
The timeline of various cell deaths

Cell death is a fundamental physiological process to maintain homeostasis, while it is also an abnormal pathological response to harmful stimuli. Traditionally, the concept of cell death is based on the morphology of dying cells, in equal to apoptotic cell death and necrotic cell death for a long time, which is also known as apoptosis and necrosis, respectively [1]. However, decades later, various kinds of cell death have sprung up, such as autophagy [2–4], pyroptosis [5, 6], PANoptosis [7, 8], necroptosis [9], ferroptosis [10, 11], cuproptosis [12, 13], parthanatos [14, 15], and alkaliptosis [16, 17] (Fig. 1). The expansion on morphological and biochemical features has enriched the definition of cell death. Pyroptosis is a gasdermin-mediated programmed cell death (PCD), which is closely related to inflammatory reaction [18]. Similar to apoptosis, it is also a kind of caspase-driven PCD but triggered by inflammasomes [19]. Additionally, the process inducing cell rupture and the release of cellular contents is consistent with necrosis to some extent [20].

Currently, pediatric cancers are still the driving causes of death for children and adolescents [21–23]. However, the underlying pathogenesis of most pediatric neoplasms remains largely unelucidated [24]. Due to the safety requirements and considerations for therapies in pediatric cancers, treatment strategies and drugs are far too scarce [25]. According to FDA database, only around 40% of children with cancer living in most developing countries tend to survive five years after initial diagnosis. Meanwhile, only eleven drugs have been approved for anticancer therapy in underage from 1980 to 2017 [26, 27]. Therefore, there is an urgent need in pediatric cancers.

Tumor cells tend to progressively evade the cell death pathways, which exert as protective mechanisms to remove damaged cells [28]. Classical forms of cell death, including apoptosis and necrosis, are important anticancer defense mechanisms in tumor killing, which have been extensively explored [1, 29]. Recently, mounting evidences have shown that pyroptotic inflammasomes and the relevant cytokines can affect tumorigenesis such as metastasis, proliferation, and invasion [30–32]. Related pathways and targets have shed light on the potential role of pyroptosis in cancer therapeutics among virous pediatric cancers like osteosarcoma [33–36], neuroblastoma [37–39], etc. However, uncertainties in treatment efficacy and the pyroptosis-mediated adverse effects reveal that the relationship between pyroptosis and pediatric cancers is not fully understood at present. Hence, we aim to focus on the molecular mechanisms, the virous functions and potential clinical applications or challenges of pyroptosis in multiple pediatric malignancies. In addition, we summarized a number of findings in order to raise awareness of pediatric cancers and identify dozens of potential cancer treatment candidates as well.

## Overview of pyroptosis

### Pyroptosis and cell death

Cell death is a common phenomenon in prokaryotic and eukaryotic cells. With the persistent progression in the field, novel signal transduction modules and inventive detection of the pathophysiological relevance have updated the classification of cell death. As the research hotspot, pyroptosis is a type of PCD that critically depends on the formation of plasma membrane pores by members of the gasdermin protein family. It is known that pyroptosis can induce inflammation with stimuli of toxins, chemotherapy drugs, and bacterial, and viral pathogens [40–43]. Termed as “pyroptosis”, it is composed of two parts originated from the Greek roots “*pyro*” and “*ptosis*”, which means “fire” and “falling”, respectively [44]. The most characteristic part of pyroptosis is the inflammatory reaction when compared with other forms of cell death. Generally, cleavage of gasdermin, leakage of interleukin (IL-1β/IL-18), bubble-like protrusions on cell membrane are typical cellular morphological features of pyroptosis, thus forming pores in the plasma membrane and allowing water to flow into the cells, and consequently causing cell swelling and lysis [45]. This process is diverse from necrosis with an explosive rupture of the plasma membrane. In contrast to fragmented nucleus in apoptosis, nuclear integrity is maintained in pyroptosis [46, 47]. DNA fragmentation is the same characteristic of pyroptosis and apoptosis, but ordered in apoptosis while random in pyroptosis [46, 48].

**Fig. 2:**
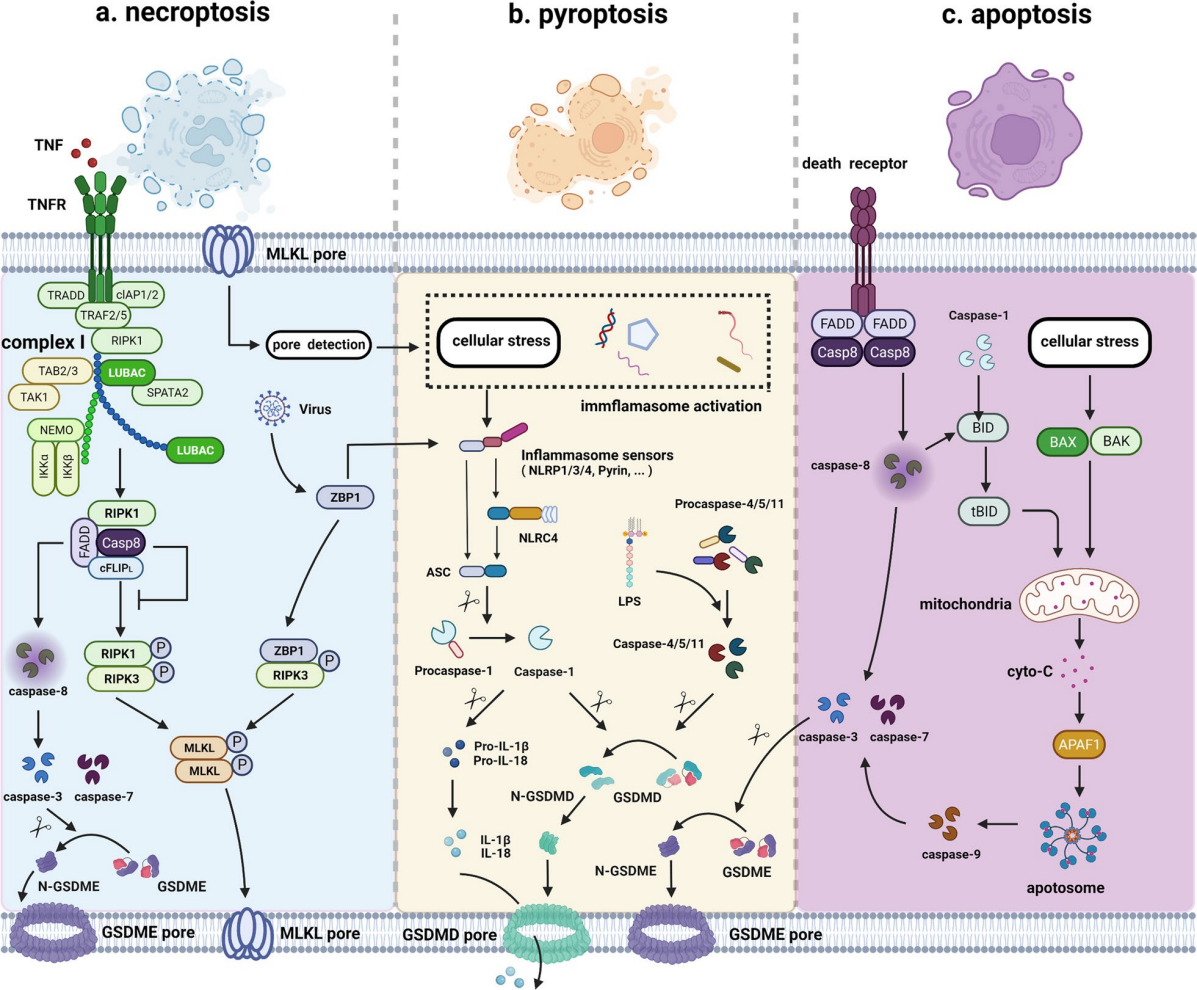
The cross talk between pyroptosis and apoptosis or necroptosis. **a–b** Interplay between necroptosis and pyroptosis. MLKL is the terminal executioner of necroptosis, which is also a key intermedium between necroptosis and pyroptosis. RIPK1-RIPK3 association or cytosolic ZBP1 activation results in phosphorylation of MLKL, thus forming pores on the membrane and engaging necroptosis. Plasma and K^+^ efflux mediated by MLKL can ultimately lead to cellular stress, triggering NLRP3 activation, inflammasome assembly, and caspase-1 cleavage, which is the canonical pyroptosis pathway. ZBP1 can directly activate NLRP3 inflammasome in response to virus infection. Additionally, when TNF binding to TNFR on the cell membrane, complex I is assembled and activated, further forming ripoptosome complex. Caspase-8 from ripoptosome complex can in turn promote the initiation of caspase-3 and -7 to execute GSDME-mediated pyroptosis. **b**–**c** Interplay between pyroptosis and apoptosis. Caspase family of proteases and its targeting downstream molecules connect apoptosis with pyroptosis. In extrinsic apoptosis, recruitment of FADD and caspase-8 promotes the initiation of the death-inducing signaling complex (DISC) when death receptor is activated. Then, activated caspase-8 from DISC can promote the initiation of caspase-3 and -7 and execute GSDME-mediated pyroptosis. In intrinsic apoptosis, Bcl-2 family member Bid can be cleaved by caspase-8 and pyroptosis-inducing caspase-1 into proapoptotic tBID, together with intracellular stress, mitochondrial outer membrane permeabilization (MOMP) is induced, subsequently triggering cytochrome c release, apoptosome formation and caspase-9 activation, which in turn promotes activation of caspase-3 and -7

The modality of cell death can be divided into two categories, namely the accidental cell death (ACD) and regulated cell death (RCD), according to the speed at which cell death occurs and its potential control by drugs or genes [1, 49]. In general, PCD is completely physiological forms of RCD. As a novel form of PCD, the morphological changes of pyroptosis are distinctive from other classical forms of cell death. However, pyroptosis still holds some features that are consistent with apoptosis or necrosis, just like a mixture of the two to some extent [50, 51]. Similar to pyroptosis, necroptosis is also a lytic and inflammatory type of PCD that requires the membrane damaging proteins, but in a caspase-independent fashion mainly mediated by ﻿receptor-interacting protein 1 (RIP1), RIP3, and mixed lineage kinase domain-like (MLKL) [20, 52] (Fig. 2). Notably, due to the extensive cross talk among pyroptosis, apoptosis and necroptosis, the concept of PANoptosis is established. PANoptosis is an inflammatory PCD pathway with key features of pyroptosis, apoptosis, and/or necroptosis, although differences exist in key initiators, effectors, and executioners in each of these three PCD pathways [7, 53–55]. Ferroptosis is classified as regulated necrosis that is characterized by iron-dependent lipid peroxidation and contains various biologic processes like lipid metabolism, iron metabolism, oxidative stress, and biosynthesis of nicotinamide adenine dinucleotide phosphate (NADPH), glutathione (GSH), and coenzyme Q10 (CoQ10) [56–58]. More recently, excess concentration of copper is currently conformed to mediate a distinct form of cell death, which is dependent on mitochondrial respiration and protein aggregation [12, 59]. Pyroptosis is an inflammatory form of PCD, while autophagy is a subcellular process that plays an important role in maintaining homeostasis when degradation of proteins and damaged organelles occur [60, 61]. Taken together, the comparisons of the characteristics among these different forms of PCD are briefly summarized in Table 1.

**Table 1:** Comparison of known programmed cell death

Type	Cellular morphological features				Biochemical features				Immune features	
	Morphology	Membrane	Organelle	Remark(s)	DNA	Pore-forming cause	*	Caspase-dependent	Inflammation	Remark(s)
Pyroptosis	Swell	Pore formation	Intact/deformed	Inflammasome	RD	Gasdermin protein	√	√	√	ICD
Apoptosis	Shrink	Intact	Intact	Apoptotic body	LD	No	×	√	×	TCD or ICD
Necroptosis	Swell	Pore formation	Swell	Necrosome ^#^	RD	MLKL	√	×	√	ICD
Autophagy	Crescent-shaped	Intact	Engulfed by autophagosome	Autophagosome	RD	No	×	×	×	ICD
Ferroptosis	Swell	Pore formation	Smaller mitochondria	Autophagosome	RD	Iron-dependent lipid peroxidation	√	×	√	ICD
PANoptosis	NA	Pore formation	NA	PANoptosome	RD	Gasdermin/MLKL	√	√	√	TCD or ICD
Parthanatos	Not swell	Loss integrity	NA	Chromatin condensation	Large DNA fragmentation	NA	NA	×	√	ICD
Alkaliptosis	Necrosis-like	Pore formation	swell	Alkalinization-dependent	NA	NA	NA	×	√	ICD

“ *” indicates “IL-18, IL-1b release”; “ ^#^” indicates “a key molecular signaling platform in necroptosis primarily comprising RIPK1 and RIPK3”; ICD: immunogenic cell death; IL: interleukin; LD: ladder degradation; MLKL: mixed lineage kinase domain-like; NA: not available; RD: random degradation; RIP: receptor-interacting protein; TCD: tolerogenic cell death

### Molecular mechanisms of pyroptosis

The formation of extracellular or intracellular stimulation and inflammasome is the initial process of pyroptosis [74, 75]. Afterward, virous inflammasomes act as platforms for the activation of caspases, which subsequently starts or executes cellular programs [1, 76]. Triggered by different caspases, the pathways associated with pyroptosis can be generally divided into canonical pathway, noncanonical pathway and other pathways, including caspase-3/8-mediated pathway and granzyme-mediated pathway [77]. The detailed pyroptotic pathways are depicted as follows:

#### Canonical pyroptotic pathway

Canonical pyroptosis is mediated by caspase-1, with inflammasome assembly, gasdermin D (GSDMD) cleavage and interleukin release (mainly IL-1β and IL-18) [18, 20, 78]. Pattern recognition receptors (PRRs) like Toll-like receptors (TLRs) and nod-like receptors (NLRs) recognize intracellular and extracellular signals, mainly danger-associated molecular patterns (DAMPs) and pathogen-associated molecular patterns (PAMPs), then initiate a signaling cascade which leads to pro-inflammatory cytokines release and GSDMD-mediated cell death [79, 80]. The canonical inflammasome can be assembled by inflammasome sensors like nod-like receptor protein 1 (NLRP1), NLRP3, NLR family CARD domain-containing protein 4 (NLRC4), AIM2, and pyrin [77], thus detecting diverse microbial or intracellular danger signals and activating caspase-1 [29].

**Fig. 3:**
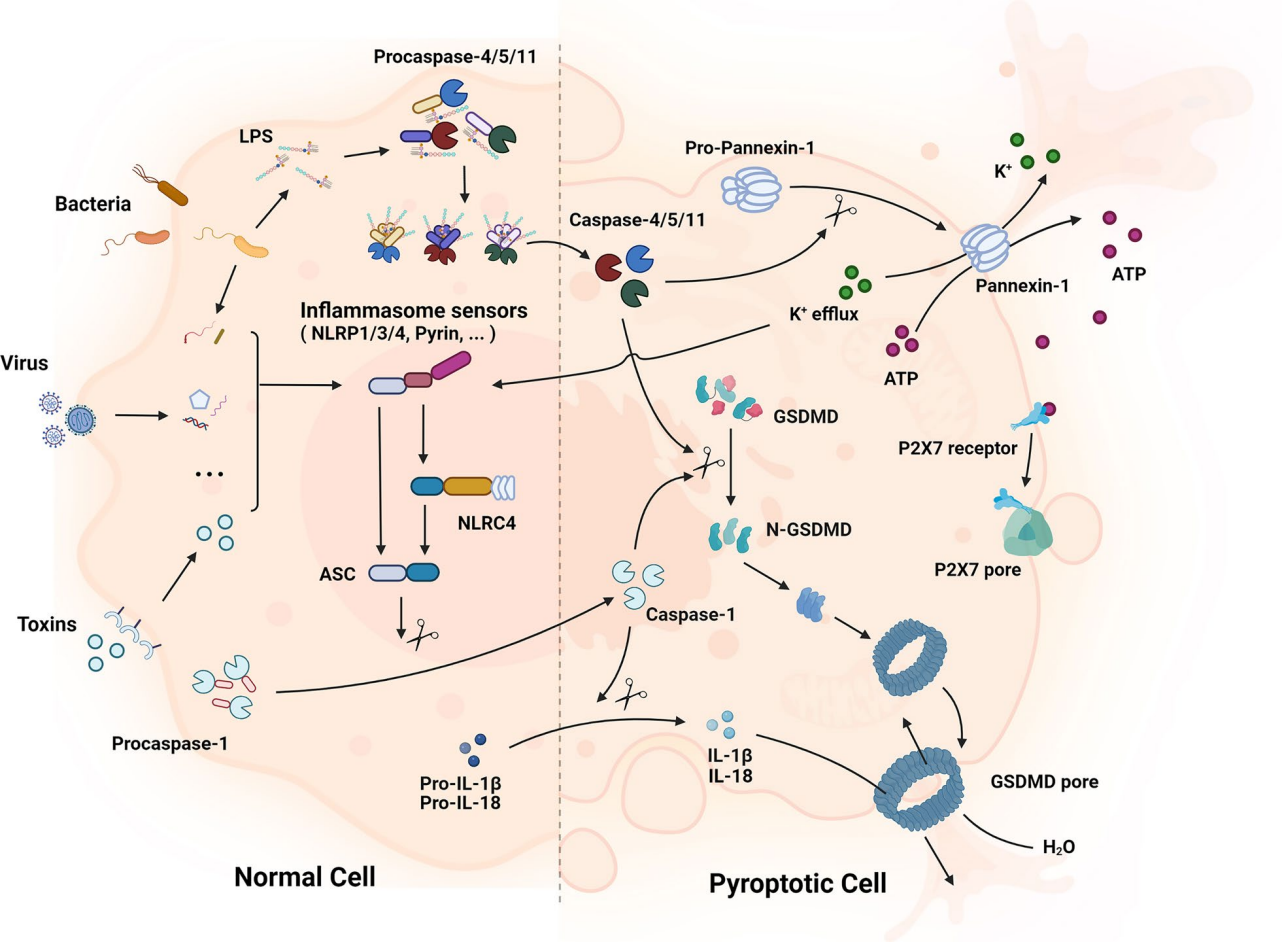
The canonical and noncanonical pathway of pyroptosis. In the canonical pathway, PRRs like TLRs and NLRs recognize intracellular and extracellular signals such as DAMPs and PAMPs; then, they assemble with pro-caspase-1 and ASC to form inflammasomes and active caspase-1. Afterward, GSDMD and pro-IL-1β/18 are cleaved into N-GSDMD and IL-1β/18. N-GSDMD perforates the cell membrane by forming nonselective pores, and IL-1β and IL-18 are secreted from the pores, eventually resulting in cell swelling and lysis. In the noncanonical pathway, cytosolic LPS activates caspase-4/5 in human and caspase-11 in mice, respectively. Then, with a process of GSDMD cleavage, cell membrane pores formation, and osmotic cell lysis, pyroptosis is forming. Additionally, the activated caspase-11 can cleave pannexin-1, resulting in the release of ATP and P2X7-mediated pyroptotic cell death

At the beginning, expressions of inflammasome components, including NLRP3, caspase-1 and pro-IL-1β, are increased with an up-regulation of nuclear factor-kB (NF-kB) [81, 82]. Then, inflammasome sensors like NLRP3 are activated via stimulation of various pathogenic signals induced by numerous PAMPs or DAMPs like lysosomal disruption [83], K^+^ efflux [84], Ca^2+^ flux [85], etc. The inflammasome sensors assemble with pro-caspase-1 and apoptosis-related speck-like protein (ASC) afterward, to form inflammasomes and activate caspase-1. Activated caspase-1 cleaves GSDMD into N-terminal domain of GSDMD (GSDMD-N), which leads to nonselective pores forming on cell membrane, and eventually results in cell swelling and lysis. It also induces the conversion of pro-IL-18 and pro-IL-1β into mature inflammatory cytokines, hence further promoting the transcriptional activities of NF-kB and numerous factors in other inflammatory and stress-induced pathways [82, 86] and thereby forming a positive feedback loop (Fig. 3).

#### Noncanonical pyroptotic pathway

Noncanonical pyroptotic pathway is simpler and more straightforward compared with the former, which is triggered by the activation of caspase-4/5 in human and caspase-11 in mice, respectively [76, 77]. Noncanonical inflammasome sensors can directly detect intracellular bacteria and lipopolysaccharide (LPS), thus activating caspase-4/5/11. Later, with a process of GSDMD cleavage, cell membrane pores formation, and osmotic cell lysis, pyroptosis is forming. It is worth noting that ion influx and efflux play a significant role in this process. Cleavage of GSDMD results in efflux of K^+^ [18, 87], which can not only lead to unbalanced osmotic pressure and water influx, but can also help mediate the assembly of NLRP3 inflammasome [86, 88, 89], thus promoting pyroptosis. Intriguingly, caspase-4/5/11 can only mediate the maturation and secretion of IL-1β/ IL-18 in canonical caspase-1 pathway without directly cleaving them [29]. Additionally, caspase-11 is conformed to cleave pannexin-1 followed by cellular adenosine triphosphate (ATP) release, purinergic P2X7 pathway activation, and eventually lead to cytotoxicity or cytolysis [90], which is a significant supplement to noncanonical pyroptotic pathway (Fig. 3).

#### Other pathways

**Fig. 4:**
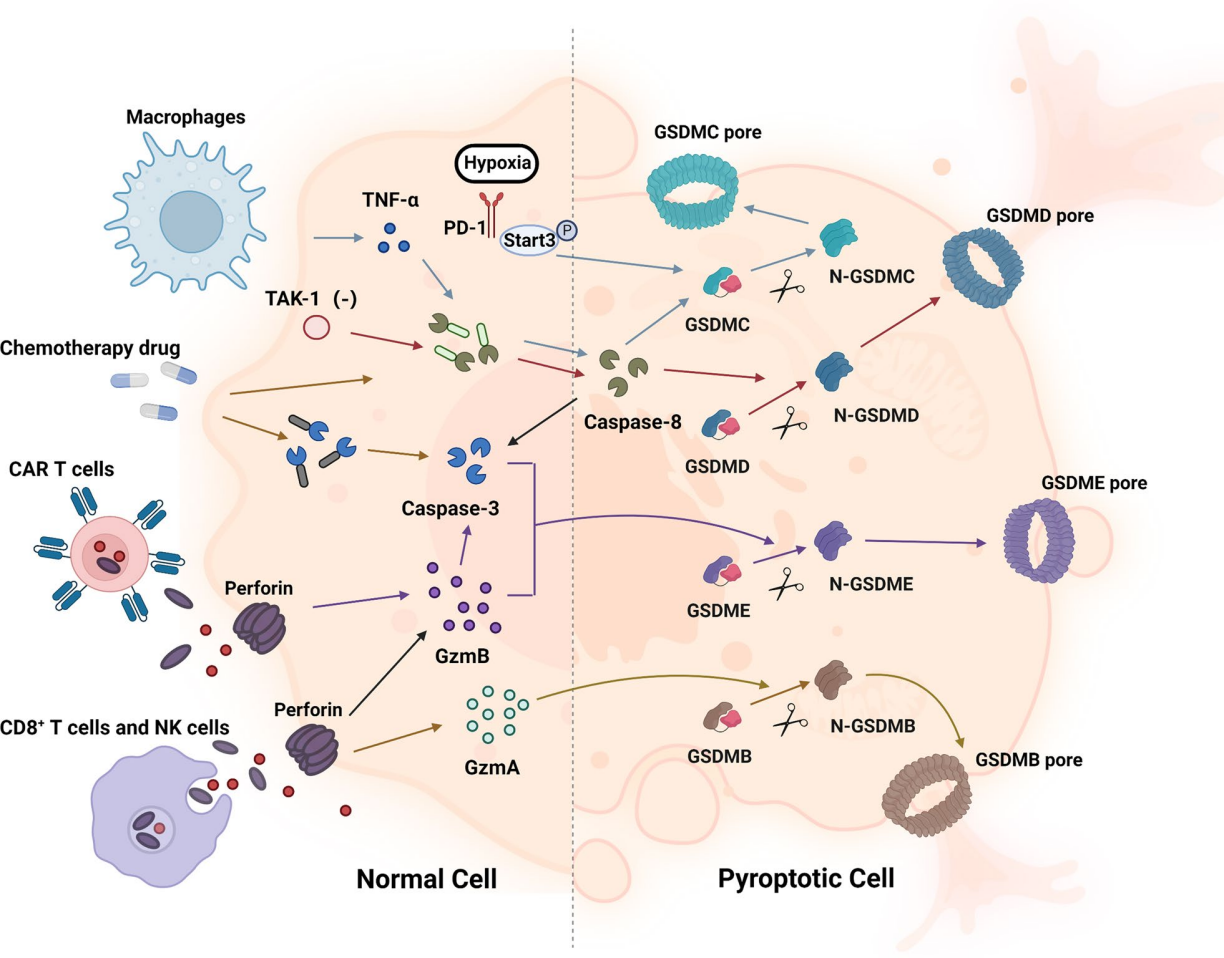
Other pathways of pyroptosis. In the caspase-3/8-mediated pathway, the inhibition of TAK1 activates caspase-8, resulting in GSDMD cleavage and pyroptosis. With the help of p-Stat3, PD-L1 is transferred to the nucleus and upregulates the transcription of GSDMC under hypoxia conditions. Activated by TNF-α, caspase-8 specifically cleaves GSDMC into GSDMC-N and eventually forms pores on the cell membrane, causing cell swelling, lysis and death. Chemotherapeutic drugs could induce caspase-3-mediated GSDME cleavage with high GSDME expression and form N-GSDME termini, which caused pyroptosis of tumor cells. In the granzyme-mediated pathway, CAR-T cells activate caspase-3 in target cells and release GzmB, causing GSDME-mediated pyroptosis, while GzmA secreted from CD8^+^ T cells and NK cells induces GSDMB-mediated pyroptosis

At present, caspase-3/8-mediated pathway and granzyme-mediated pathway have been reported (Fig. 4). The former one is triggered by caspase-3/8, which is previously considered as the exclusive process in apoptosis. The view was expanded with the discovery of caspase-3-mediated gasdermin E (GSDME) cleavage in tumor cells induced by chemotherapeutic drugs [62], and caspase-8-related cleavage of GSDMD in mouse macrophages during Yersinia infection [91]. Interestingly, tumor necrosis factor (TNF)-mediated apoptosis is converted into pyroptosis with the expression of gasdermin C (GSDMC) mediated by programmed cell death ligand 1 (PD-L1) in breast cancer [92]. Under hypoxia conditions, PD-L1 is transferred to the nucleus and with the help of p-Stat3, and they together upregulate the expression of GSDMC. Later, caspase-8 specifically cleaves GSDMC and eventually induces pyroptosis [92, 93]. Additionally, chemo-antibiotic drugs like daunorubicin, actinomycin D, doxorubicin (DOX), and epirubicin were found to increase the expression of GSDMC and nuclear PD-L1 in breast cancer, which further promoted caspase-3/8-mediated pathway and ultimately led to pyroptosis [92, 93].

Granzyme-mediated pathway further broadens the definition of pyroptosis, which was previously known to be only activated by caspases [94, 95]. Studies found that chimeric antigen receptor-T (CAR-T) cells could activate caspase-3 in target cells and release granzyme B (GzmB), subsequently causing GSDME-mediated pyroptosis, while granzyme A (GzmA) secreted from CD8 + T cells and NK cells could induce gasdermin B (GSDMB)-mediated pyroptosis [95, 96].

### Pyroptosis in common pediatric cancers

**Fig. 5:**
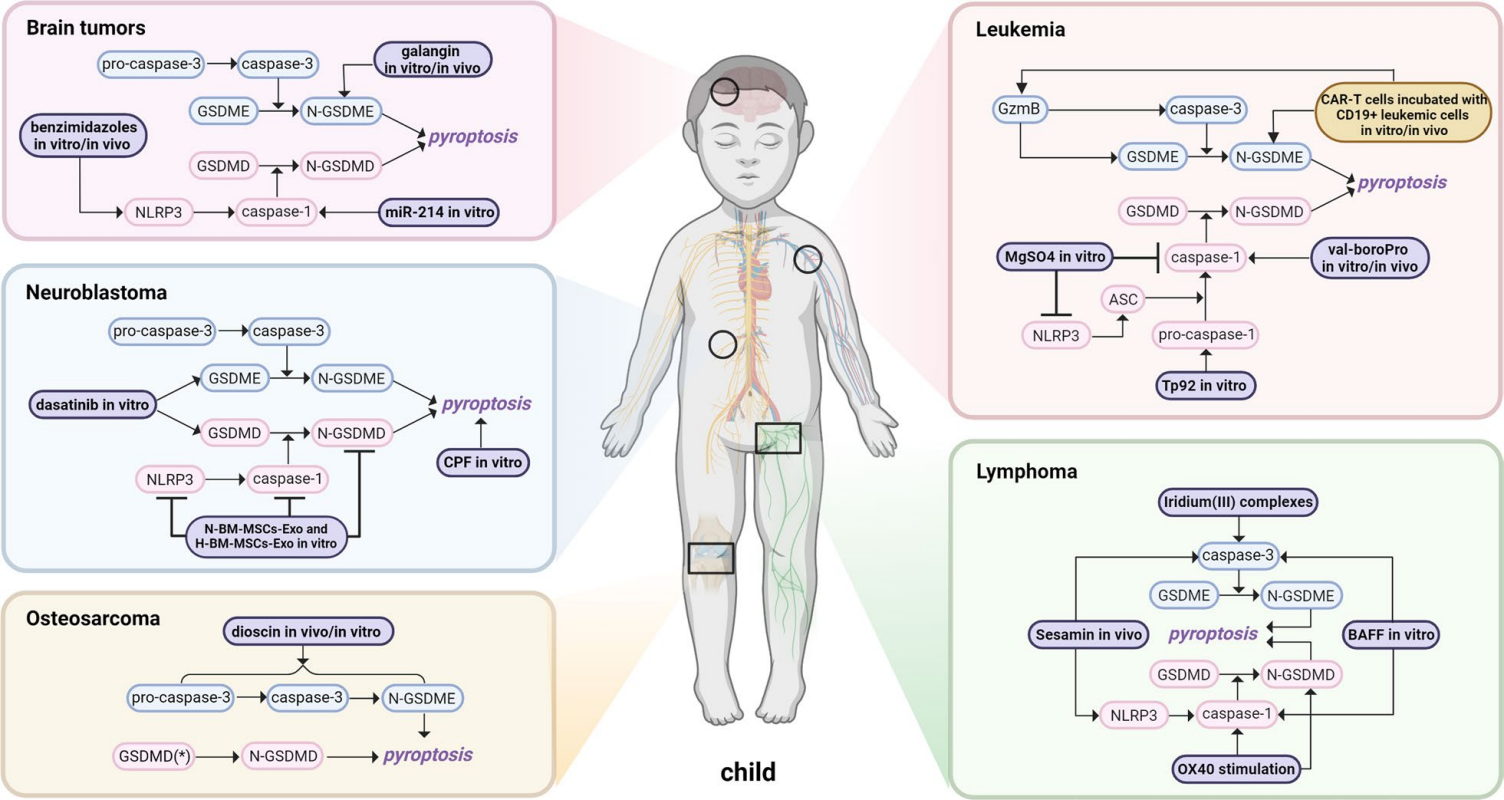
Mechanisms of pyroptosis across several common pediatric cancers, including osteosarcoma, neuroblastoma, leukemia, lymphoma, and brain tumors

Here, we discussed the recent advances concerning the underlying molecular mechanisms of pyroptosis and the potential challenges for regulating pyroptosis pathways through various oncologic therapies in several common pediatric cancers, including osteosarcoma, neuroblastoma, leukemia, lymphoma, and brain tumors. The detailed findings are shown in Table 2 and Fig. 5.

**Table 2:** Roles of pyroptosis across various pediatric cancers

Cancer types	Design	Experimental design	Cellular process	Cell viability and impact
Osteosarcoma	Dioscin	In vitro*/*in vivo	G2/M-phase arrest, apoptosis, and GSDME-dependent pyroptosis	Inhibited cell proliferation
	Clinical control and statistical analysis	Primary clinical samples and normal bone tissues	NA	Poor chemotherapy response, distant metastasis, worse prognosis
Neuroblastoma	Dasatinib	In vitro	Pyroptosis	Low cell survival rate
	CPF	In vitro	NLRP3-dependent pyroptosis via miR-181/SIRT1/PGC-1α/Nrf2 pathway	Inhibited cell viability, and proliferation
	BM-MSCs-Exo	In vitro	OGD-mediated pyroptosis, inhibited GSDMD shuttle from nucleus to cytoplasm	Reduced dead cell ratio
Leukemia	Val-boroPro	In vitro*/*in vivo	Pro-caspase-1-dependent pyroptosis	Reduced cell viability, and increased cell death ratio
	MgSO4	In vitro	Inhibited ASC oligomerization, NLRP3-dependent pyroptosis	Smaller cell size, lower level of pyroptotic cell death
	Tp92	In vitro	Atypical pyroptosis via pro‐caspase‐1 pathway, apoptosis via the RIPK1/caspase‐8/caspase‐3 pathway	Increased cell death ratio
	CAR-T cells incubated with CD19 + leukemic cells	In vitro*/*in vivo	GSDME-mediated pyroptosis	Decreased cell viability, CRS controlled
Lymphoma	Sesamin	In vivo	Apoptosis, pyroptosis, autophagy	Inhibited growth and proliferation
	BAFF	In vitro	NLRP3-dependent pyroptosis, src activity-dependent ROS production, potassium ion efflux	Decreased cell viability
	OX40 stimulation	In vivo	Pro-caspase-1-dependent pyroptosis	Decreased cell viability, and liver injury
	Iridium (III) complexes	In vitro*	Mitochondria-mediated apoptosis, GSDME-mediated pyroptosis	Decreased cell viability
Brain tumors	Benzimidazoles	In vitro*/*in vivo	Cell cycle arrest via P53/P21/cyclin B1, mitochondria-dependent apoptosis, NLRP3-dependent pyroptosis	Inhibited cell proliferation, migration, and invasion
	Galangin	In vitro*/*in vivo	Apoptosis, pyroptosis and autophagy	Suppressed tumor growth, reduced cell viability and proliferation
	miRNA-214	In vitro	Caspase-1-mediated pyroptosis	Inhibited cell proliferation and migration

*indicates lung carcinoma, gastric adenocarcinoma, hepatocellular carcinoma, cervical cancer, and melanoma; AML: acute myeloid leukemia; CPF: chlorpyrifos; CRS: cytokine release syndrome; GSDMD: gasdermin D; GSDMD-N: gasdermin D N-terminal; GSDME: gasdermin E; GSDME-N: gasdermin E N-terminal; HMGB1: high-mobility group protein box 1; IL: interleukin; MDS: myelodysplastic syndromes; NA: not available; NLRP3: nod-like receptor protein 3; OGD: oxygen–glucose deprivation; PTX: paclitaxel; ROS: reactive oxygen species

### Osteosarcoma and pyroptosis

Osteosarcoma, a kind of malignant tumor derived from mesenchymal tissue, is highly aggressive in young populations [97, 98]. The incidence of osteosarcoma is bimodal with age, with an initial peak at 10–14 years of age, and a second peak after 60 years [99]. Primarily, osteosarcoma tends to occur in the metaphysis of the distal femur and proximal tibia [100], while metastasis is mainly detected in the lung [101, 102]. With an annual incidence of approximately 3–4 patients per million, osteosarcoma is a relatively common malignant tumor in pediatric cancers, as well as one of the leading causes of cancer-related death in children and adolescents [103, 104]. Owing to the combination of surgical resection and neoadjuvant chemotherapy, osteosarcoma is among the most curable malignant tumors of high-grade sarcomas and achieves a long-term survival rate about 70% in patients with localized disease [105, 106]. However, patients still suffer from unsatisfactory clinical outcomes including physical disability, drug resistance and other serious adverse effects [107, 108], especially in patients with pulmonary metastasis or local relapse. This also reflects the slow progress of osteosarcoma treatment paradigms, treatment satisfaction and survival rate over the past few years. Therefore, the identification of novel therapeutic agents and treatment strategies is urgently needed.

Recently, emerging researches have focused on different targets in the pyroptosis pathways and investigated the regulatory role and molecular mechanisms of pyroptosis among different models, which have brought mounting promising candidates for the osteosarcoma treatment. Inspired by traditional Chinese medicine, Ding et al. found that dioscin (a steroidal saponin derived from medicinal plants) inhibited the growth of human osteosarcoma both in vitro and in vivo [33], demonstrating the anticancer potential of dioscin in osteosarcoma. Dioscin could inhibit osteosarcoma cell proliferation and induce G2/M-phase arrest and apoptosis in a concentration-dependent manner. Compared with the control, shrinkage, chromatin condensation, and nuclear fragmentation were more frequently observed in osteosarcoma cells exposed to dioscin. Additionally, involvement of caspase-3-GSDME-N axis in this process implicated by mechanistical analysis further suggests that dioscin could induce pyroptosis via cleavage of GSDME and formation of GSDME pore on the cancer cell membrane [33]. Besides, one GSDMD-targeted statistical analysis showed the GSDMD was significantly overexpressed in osteosarcoma compared to non-neoplastic counterparts, and the elevated expression of GSDMD was obviously associated with poor chemotherapy response, distant metastasis, and worse prognosis of osteosarcoma patients [34]. It is known that pyroptosis may result in the cleavage of GSDMD and activation of cytokines such as IL-1β and IL-18 [1]. It is estimated that upregulated GSDMD expression might play a considerable role in the progression and treatment resistance of osteosarcoma, which was already observed in other carcinomas, including nonsmall cell lung cancer [109], bladder cancer [110], and breast cancer [111].

Currently, there are few in vitro or in vivo studies concerning the role of pyroptosis in osteosarcoma, but the related prognostic analysis may also have some implications in evaluation of both treatment and prognosis. Zhang et al. identified a novel pyroptosis-related gene signature to predict the prognosis and indicate the features of immune microenvironment of patients with osteosarcoma [36]. Six pyroptosis-associated risk genes were identified via univariate and LASSO regression analysis. Combined with other clinical characteristics, an independent pyroptosis-related prognostic factor named "PRS-score" for osteosarcoma patients was established, which might be an important contribution to early diagnosis and prognosis of immunotherapy in osteosarcoma [36]. Similarly, another study constructed three differentially expressed pyroptosis-related long noncoding RNA (lncRNA) signature linked to osteosarcoma microenvironment and prognosis [35], which had critical predictive value for the prognosis of osteosarcoma patients and may be promising targets for future therapy.

### Neuroblastoma and pyroptosis

Neuroblastoma, mainly affecting the normal development of the paravertebral sympathetic ganglia and adrenal medulla, is the most common extracranial embryonal malignancy in children [112]. Different locations of the tumor and paraneoplastic syndromes contribute to variable manifestations of neuroblastoma, leading to dramatic prognosis ranging from spontaneous tumor regression to widespread metastasis, which is unresponsive to treatment [113, 114]. Children at high risk of neuroblastoma metastasis or mortality receive multimodality treatment, but may suffer from complications like nausea and pain. *MYCN* gene amplification has been confirmed to be in line with high-risk cases of neuroblastoma [112]. Tremendous efforts to develop MYCN inhibitors with undesirable outcomes indicate that this nuclear transcription factor may be difficult to target therapeutically. Aiming at better cures and long-term quality of life for children with cancer, identification of novel immunotherapy agents and tumor targets are underway with several promising approaches like ^123^I-MIBG imaging followed by autologous stem cell rescue [115], lutetium 177-DOTATATE [116], and activating mutations in the genes of RAS-MAPK pathway [117], etc. However, breakthrough is still far from reach.

Recent advances in pyroptosis research have cast a light in the dark. Gasdermins are typical proteins involved in pyroptosis, and their cleavage fragments can insert into the cell membrane and thus play different roles in cytolysis. GSDME, participating in chemotherapy-induced pyroptosis in tumor cells, is promoted by anti-oncogene p53 [62, 118]. But continuous expression of p53 leads to apoptosis in normal cells after treatment with cytotoxic anti-tumor agents [119]. Zhang and colleagues reported that dasatinib could induce pyroptosis in neuroblastoma SH‑SY5Y cells and increase the protein levels of GSDMD and GSDME out of the effect of p53 [38]. It is of significant importance in therapy efficacy of neuroblastoma. Pyroptosis caused by small molecule toxicants has been reported in several studies like athiopyran derivative with low murine toxicity [120], and bilirubin mediated toxicity in cultured rat cortical astrocytes [121], etc. Interestingly, one study showed that the level of pyroptosis-related proteins was significantly increased in a dose and time-dependent manner in SH‑SY5Y cells when treated with insecticide chlorpyrifos (CPF) [39]. By upregulating miR-181 through downregulation of the SIRT1/PGC-1α/Nrf2 pathway, CPF promotes cell pyroptosis, inhibits cell proliferation and increases susceptibility to oxidative stress-induced toxicity. But the active ingredients and toxicity of CPF should be considered in therapeutic treatment.

In addition of anti-tumor effect, the role of pyroptosis on noncancerous tissue damage has been investigated as well. For example, Kang et al. reported the neuroprotective effects of bone marrow mesenchymal stromal cells-derived exosomes (BM-MSCs-Exo) under normoxic and hypoxic conditions, which named N-BM-MSCs-Exo and H-BM-MSCs-Exo, respectively. The research was investigated on oxygen–glucose deprivation (OGD) injury in mouse neuroblastoma N2a cells and rat primary cortical neurons [37]. Functional assays and mechanistical analysis showed that pyroptosis-related proteins were decreased in both groups, demonstrating that hypoxic BM-MSCs-Exo may have a more pronounced protective effect in ameliorating the progression of cerebral ischemia and hypoxia injury in patients.

### Leukemia and pyroptosis

Leukemia is a common pediatric cancer characterized by abnormal excessive proliferation of hematopoietic stem cells in bone marrow or blood [122, 123]. It can be mainly divided into two categories: acute lymphocytic leukemia (ALL) and acute myeloid leukemia (AML), among which ALL is more prevalent [124, 125].

As a novel inflammasome sensor, caspase recruitment domain-containing protein 8 (CARD8) can bind to pro-caspase-1 [126, 127], and it was confirmed to trigger pyroptosis in myeloid leukemia cells through inhibition of dipeptidyl peptidases (DPP) [128]. Nonselective DPP-inhibitor val-boroPro (also called PT-100 and talabostat) is a common anticancer drug, which can inhibit DPP8/9, thus inducing pyroptosis in a NLRP1b-dependent manner in myeloid cells [127]. Further studies showed that val-boroPro could induce pyroptosis in multiple kinds of AML cell lines in vitro, and inhibit the AML progression in vivo. Taken together, inhibition of DPP may be a novel therapeutic strategy for AML [127, 129]. It is known that calcium signaling is important in the activation of the NLRP3 inflammasome [130, 131]. As a common calcium antagonist, MgSO4 could inhibit the release of calcium-influx-dependent histamine [132], which enables its potential anti-inflammatory effect. Based on this, Chang et al. found that MgSO4 could downregulate NLRP3 inflammasome, caspase-1 and IL-1β in THP-1 cells (human monocytic cell line derived from an acute monocytic leukemia patient), thus inhibiting NLRP3-dependent pyroptosis [133]. Additionally, another study proved that Tp92, the only outer membrane protein of Treponema pallidum [134] could induce human mononuclear cell death and IL-8 secretion. And interestingly, among the various mechanism, Tp92 may induce atypical pyroptosis of THP-1 cells via the pro-caspase-1 pathway [135]. However, it is worth noting that the production of IL-1β and IL-18 is absent in this process, which is commonly seen in canonical pathway of pyroptosis. As known, CAR-T cell therapy is a great success in clinical applications of genetically engineered T cells modified with CARs against B cell malignancies, but cytokine release syndrome (CRS) significantly restricts its effectiveness and extensibility [136, 137]. A study by Liu et al. reported that upon incubation with CD19 + leukemic cells, CAR-T cells could increase the release of lactic dehydrogenase (LDH), and upregulate expression GSDME and IL-1β, suggesting that CAR-T cells can activate GSDME-mediated pyroptosis by releasing a large amount of perforin and GzmB, and could ultimately result in CRS [94]. Further study also revealed that CRS occurrence significantly decreased when depleting macrophages, knocking out GSDME, or inhibiting caspase-1 in mouse models [94]. Since high expression of GSDME is observed in B leukemic cells, the induction of CRS may impede the application of pyroptosis related CAR-T therapy in leukemia. Therefore, studies focusing on other forms of PCD may be alternative promising choices for CAR-T therapy.

### Lymphoma and pyroptosis

Lymphoma is a common but highly treatable malignancy in children [138]. It can be further divided into Hodgkin lymphoma (HL) and non-Hodgkin lymphoma (NHL) [139]. Accounting for about 5% of childhood cancers, NHL is more likely to occur in younger children when compared with HL. However, NHL is still uncommon in children younger than 3-year-old [140]. NHL may develop from either abnormal B or T cells, while HL derives from a specific abnormal B lymphocyte lineage [141].

Sesamin is a plant-derived compounds with many pharmacological effects including antiproliferative, antimetastatic, anti-inflammatory, and proapoptotic functions [69, 142, 143], which makes it a promising candidate for anticancer treatment. It is reported that sesamin markedly inhibited growth and proliferation of EL4 cells by inducing apoptosis, pyroptosis and autophagy to strengthen the anti-tumor effects on murine T cell lymphoma [144]. B cell-activating factor (BAFF), a member of TNF superfamily, supports B cell survival and homeostasis through the activation of the NF-*κ*B pathway [145]. It is well known that NF-*κ*B is a key initiating signal of NLRP3 inflammasome [146], but the relationship between BAFF and NLRP3 inflammasome remains unclear. Lim et al. firstly demonstrated BAFF induced the activation of NLRP3 inflammasomes with increased expression of NLRP3, IL-1β, and caspase-1, and ultimately leading to pyroptosis in primary B cells and B lymphoma cell lines [147]. Coincidentally, another TNF superfamily receptor OX40 (also called CD134) was found to trigger the activation of caspase-1, resulting in IL-1β expression as well as the cleavage of the pyroptotic protein GSDMD in invariant nature killer T (iNKT) cells [148]. Of note, iNKT cells mainly reside in liver, more researches on other kinds of T cells may uncover the clinical implications in the development of OX40-directed therapies in lymphoma. Interestingly, Zhang et al. reported a new ligand TFBIP (2-(4’-trifluoromethyl)-[1,1’-biphenyl]-4-yl)-1H-imidazo[4,5-f] [1, 10] phenanthroline) and its three iridium (III) complexes [70]. When trapped in liposomes, these complexes can trigger mitochondria-mediated apoptosis and GSDME-mediated pyroptosis in a variety of cancers, including lung carcinoma, gastric adenocarcinoma, and melanoma. More work in progress identifying the effect of these complexes in lymphoma may help bring potential anticancer strategies about TFBIP in this malignancy in the future.

### Brain tumors and pyroptosis

Primary tumors of central nervous system (CNS) are the most frequent solid tumors in children, contributing to about 15% to 20% of all malignancies in pediatrics [149]. Pediatric high-grade glioma (pHGG) is one of the rapidly lethal malignancies at young age [150, 151]. Among them, glioblastoma, also known as glioblastoma multiforme (GBM), is the most prevalent and aggressive [152]. To date, surgical resection followed by adjuvant radiation therapy and chemotherapy with oral temozolomide (TMZ) is the most common and effective treatment for GBM patients [153]. However, TMZ, the only drug available for GBM, frequently induces drug resistance and numerous side effects [154].

A study by Ren et al. found benzimidazoles can induce cell cycle arrest at the G2/M phase via the p53/p21/cyclin B1 pathway, and concurrently induce mitochondria-dependent apoptosis and NLRP3-dependent pyroptosis in glioblastoma cells [30]. Another study revealed a natural flavonoid Galangin could also elicit a potent anti-tumor effect on GBM by initiating apoptosis, pyroptosis and autophagy [67]. These novel drugs cater to the urgent clinical need for GBM therapeutics especially in patients who are resistant or less responsive to TMZ. MicroRNAs (miRNAs) are small endogenous noncoding RNAs with a variety of targets, and thus exerting diverse functions in tumorigenesis, progression, and metastasis [155–157]. In glioma, an inverse relationship between abundant caspase-1 expression and low expression of miRNA-214 was observed [158, 159]. Following this, another research further confirmed caspase-1 as a target gene of miRNA-214 by luciferase reporter assay [71]. Moreover, miRNA-214 was found to inhibit cellular proliferation and migration via caspase-1 dependent pyroptosis in glioma [71]. In addition to approved drugs repurposing and new drugs development, Lu et al. reported a novel strategy to package recombinant adeno-associated virus (rAAV) expressing GSDMD-N. They have successfully delivered GSDMD-N into tumor cells and demonstrated pyroptosis induced by these rAAVs in preclinical cancer models including glioblastoma [72], which can provide enlightenment for the idea of anti-tumor therapy. Till now, a growing body of research has posted attention on GBM. Exploration of novel drugs, and combination of traditional methods or medicine may also facilitate progression in pediatric brain tumors.

### Pyroptosis related therapy in pediatric cancers

**Fig. 6:**
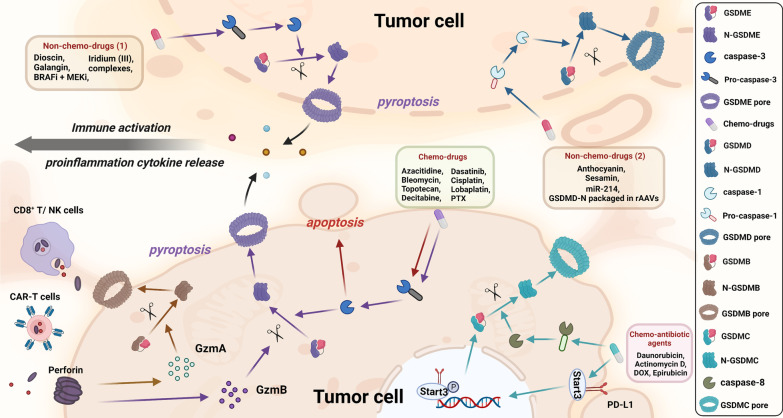
Mechanism of chemotherapy drugs and nonchemotherapy drugs in pyroptosis pathway in tumor cells. **a** Chemotherapy drugs mainly induce GSDME-mediated pyroptosis via activation of pro-caspase-3, while caspase-3 can further promote apoptosis. Formation of GSDME pore leads to cytolysis, cytokine release, and activation of immune cells like dendritic cells, CD8^+^ T cells, and NK cells. GzmA secreted from CD8^+^ T cells and NK cells induce GSDMB-mediated pyroptosis while CAR-T cells can activate caspase-3 in target cells and release GzmB, promoting GSDME-mediated pyroptosis. Chemo-antibiotic drugs help increase the expression of GSDMC and nuclear PD-L1, and with the help of p-Stat3, they together upregulate the expression of GSDMC. Later, caspase-8 specifically cleaves GSDMC and eventually induces pyroptosis. **b** Mechanisms of nonchemotherapy drugs are more complex. Drugs like dioscin, galangin, BRAFi/MEKi, etc. induce GSDME-mediated pyroptosis and release proinflammatory cytokines from pyroptotic or apoptotic pores, which could subsequently initiate the activation of immune systems. Meanwhile, other reagents like anthocyanin and sesamin mainly exert their role in pyroptosis via GSDMD pathway in tumor cells

Apoptosis resistance is a general hallmark of cancer in the mechanism of tumor drug resistance [160, 161]. Recently, treatments that target nonapoptotic cell death have attracted great attention [162, 163]. Therefore, the research in different forms of pyroptosis is of great significance for the treatment therapy of pediatric cancers (Fig. 6).

### Chemotherapy drug-induced pyroptosis

Chemotherapy is a diverse and rapidly progressing field [164, 165]. While most researches explicitly cast attention to adult diseases, considerable efforts are being made to bring these therapies into the pediatric cancers. Chemotherapy drugs can initiate caspase-3-mediated apoptosis in cancer cells, but the process will be switched to pyroptosis with abundant GSDME expression [62, 166]. As proved, GSDME is lowly expressed in most cancer cells [167–170], so we can suppose that with reversal of GSDME expression level, pyroptotic therapy may exert an important role in cancer treatment.

Decitabine and azacitidine are classical drugs to treat myelodysplastic syndromes (MDS) or AML [171, 172]. It is reported that these drugs can increase GSDME expression and sensitize the cancer cells to chemotherapy drug by inducing GSDME-mediated pyroptosis [62, 63]. Additionally, dasatinib, a multi-kinase inhibitor, can induce pyroptosis in GSDME‑expressing lung cancer and neuroblastoma, and increase the expression of GSDMD and GSDME in a p53‑independent manner [38]. Paclitaxel (PTX) and cisplatin are representative chemo-drugs for lung cancers by inducing apoptosis, and are lately found to induce GSDME-dependent pyroptosis as well [64]. Of note, compared with PTX, cisplatin induces more severe secondary pyroptosis with higher expression of GSDME-N in lung cancer, indicating cisplatin may be a better candidate for lung cancers in pyroptosis-related therapy [64]. Wang et al. and colleagues further expanded the choices of chemotherapy drugs to DOX, actinomycin-D, bleomycin and topotecan for pyroptotic treatment in lung cancer [62]. Besides, Yu et al. found that lobaplatin could induce GSDME-dependent pyroptosis in colon cancer via reactive oxygen species (ROS) elevation, c-Jun N-terminal kinase (JNK) activation and initiation of the mitochondrial apoptotic pathway [65]. As a negative regulator of protein synthesis, eukaryotic elongation factor-2 kinase (eEF-2 K) is observed highly expressed in various tumor cells and proved to exert a critical function in modulating autophagy and apoptosis in tumor cells [173, 174]. Practically, lung cancer, colon cancer, and melanoma are uncommon in children. Current mainstream researches post their focus on adult diseases, but in fact, relevant novel research results and methods may also be applicable to clinical practice.

Intriguingly, therapies that inhibit pyroptosis via gasdermins may also effectively modulate tumor progression as well. A recent study established a novel targeted nanomedicine by packaging specific anti-GSDMB antibody into HER2 breast cancer cells and investigated its anti-tumor effect both in vivo and in vitro. The results showed that the therapy could effectively inhibit tumor growth and cell migration, suppress tumor resistance, and diminish lung metastasis [175]. More studies concerning pyroptosis-related therapy in malignancies are demanded to further address this controversial issue.

### Nonchemotherapy drugs-induced pyroptosis

Chemotherapeutic drugs are commonly used to maintain the patients' condition, but adverse effects like weight loss, listlessness, and tissue damage may always occur after long-term or overdose treatment [176]. Natural products and other therapies have turned into inviting alternatives in clinical practice for its low toxicity, wide source, and human affinity [177, 178]. As aforementioned, dioscin can inhibit cell proliferation in human osteosarcoma both in vitro and in vivo via GSDME-dependent pyroptosis [33]. As a member of natural flavonoids, galangin exerts a vital role in suppressing tumor growth and reducing cell viability of glioblastoma cells by triggering GSDME-dependent pyroptosis [67], while anthocyanin promotes the cell death of oral squamous cell carcinoma (OSCC) via activation of GSDMD-dependent pyroptosis [68]. Additionally, sesamin, a widely used plant-derived compound with multi-anti-tumor pharmacological effects, can inhibit the growth and proliferation of murine T cell lymphoma in vivo by regulating apoptosis and pyroptosis [69].

Interestingly, composite or synthetic agents can also be effective in anti-tumor treatment via pyroptotic pathway [179]. A new synthesized ligand TFBIP and its three iridium (III) complexes were found to induce GSDME-mediated pyroptosis, thus decreasing cell viability in several cancers [70]. MiR-214 targeting caspase-1 or GSDMD-N packaged in rAAVs can both induce pyroptosis in brain tumor cells [71, 72]. The reagent consisting of BRAF inhibitors and MEK inhibitors (BRAFi/MEKi) is used to treat BRAF^V600E/K−mutant^ melanoma with FDA approval. Further studies found that BRAFi/MEKi can induce pyroptosis with cleavage of GSDME and release of proinflammation factors like high mobility group protein B1 (HMGB1, an inflammatory marker of pyroptosis) [73]. Collectively, the mechanisms of these compound-based therapies are concluded in Table 3 for a better understanding.

**Table 3:** Compounds inducing pyroptosis signal pathways in cancers

Classification	Compounds	Cancer types	Mechanisms of pyroptosis induction	References
Chemotherapy drugs	Decitabine/azacitidine	MDS/AML	Caspase-3/GSDME	[62, 63]
	Dasatinib	Neuroblastoma/Lung cancer	Caspase-3/GSDME	[38]
	PTX/cisplatin	Lung cancer	Caspase-3/GSDME	[64]
	DOX, Actinomycin-D, Bleomycin, Topotecan	Lung cancer	Caspase-3/GSDME	[62]
	Lobaplatin	Colon cancer	ROS/JNK/caspase-3/GSDME	[65]
	DOX	Melanoma	eEF-2K/caspase-3/ GSDME	[66]
Natural products	Dioscin	Osteosarcoma	Caspase-3/GSDME	[33]
	Galangin	Brain tumors	GSDME	[67]
	Anthocyanin	OSCC	NLRP3/caspase-1/GSDMD	[68]
	Sesamin	Lymphoma	NLRP3/caspase-1	[69]
Reagents	Iridium (III) complexes	Several cancers*	Caspase-3/GSDME	[70]
	miRNA-214	Brain tumors	Caspase-1	[71]
	GSDMD-N packaged in rAAVs	Brain tumors	GSDMD	[72]
	BRAFi/MEKi	Melanoma	GSDME/ HMGB1	[73]

*indicates lung carcinoma, gastric adenocarcinoma, hepatocellular carcinoma, cervical cancer, and melanoma; AML: acute myeloid leukemia; BRAFi: BRAF inhibitor; DOX: doxorubicin; eEF-2K: eukaryotic elongation factor-2 kinase; GSDMD: gasdermin D; GSDME: gasdermin E; HMGB1: high mobility group protein box 1; JNK: c-Jun N-terminal kinase; MDS: myelodysplastic syndromes; MEKi: MEK inhibitor; NLRP3: nod-like receptor protein 3; OSCC: oral squamous cell carcinoma; PTX: paclitaxel; ROS: reactive oxygen species

### Radiotherapy-induced pyroptosis

Radiotherapy is a common clinical treatment for local malignancies, which can release tumor antigens as an endogenous tumor vaccination event to further induce tumor infiltration of CD8 + T cells, and ultimately leads to the shrinkage of primary tumor and distal metastases [180, 181]. Radiotherapy itself can not only trigger immunogenic cell death (ICD) in tumor cells, but can also assist chemotherapy-induced ICD with a combination of other treatment [181, 182]. The ideal radiotherapy should eliminate as many tumor cells as possible without damaging the normal tissues and inducing inflammation. Combination of radiotherapy with other therapies have been well applied in clinical trials for treatments of various cancers, including head and neck cancer [183], ovarian cancer [184], and nonsmall cell lung cancer [185]. Currently, few studies accurately describe the relationship between pyroptosis and radiotherapy. As abovementioned, AIM2 is a classical inflammasome sensor involved in canonical pyroptosis pathway. Hu et al. found that AIM2 was sensitive to double-strand DNA break in the nucleus caused by ionizing radiation and chemotherapeutic agents, thus inducing inflammasome activation and subsequent pyroptosis [186]. Another study by Li et al., also demonstrated high-dose X-ray irradiation could trigger pyroptosis that modulated by connexin 43 (Cx43) in the human umbilical vein endothelial cells (HUVECs) [187], implicating a vital correlation between radiotherapy and pyroptosis.

However, it is noteworthy that a few studies have clearly shown that radiation can cause radiotherapy-related toxicity upon activation of pyroptosis, such as bone marrow inhibition and gastrointestinal tract injury [180, 186, 188]. For instance, Liu et al. reported that radiation could induce NLRP3-mediated pyroptosis in primary cultured bone marrow-derived macrophages both in vitro and in vivo [189], suggesting that targeting NLRP3 inflammasome may be useful strategy to decrease the bone marrow injury caused by radiation via pyroptosis. Besides, it is reported that 10-Gy abdominal irradiation led to oxidative stress, inflammatory reaction, and NLRP3-mediated pyroptosis, and ultimately resulted in intestinal injury in mouse model [188]. Since flagellin A N/C could effectively inhibit radiation-induced pyroptosis, and in turn alleviating the intestinal injury, it is assumed that flagellin A N/C may be a potential candidate to protect patients against ionizing radiation damage [190].

### The adverse effects of pyroptosis on tumor therapy

Tissue damage is undoubtedly the most common side effect of oncologic therapies that targeting pyroptosis [49, 191]. As previous studies showed, chemo-drugs mainly induce pyroptosis by the executioner GSDME via caspase-3 activation. Therapies that targeting cancer cells with high GSDME expression are speculated to show a promising therapeutic effect. However, it is worth noting that GSDME has been widely overexpressed in normal cell while most tumor cells tend to express low GSDME due to GSDME gene promoter methylation [62, 166]. Thus, we may observe an interesting phenomenon that tumor cells with low or no expression of GSDME underwent apoptosis after chemotherapies, while normal tissues with high GSDME expression may suffer from severe toxicity via caspase-3-mediated pyroptosis. For instance, studies found that GSDME^−/−^ mice were protected from chemotherapy-induced tissue damage and weight loss, while intraperitoneal injection of cisplatin or 5-FU caused immune cell infiltration and severe small intestinal injury in GSDME^+/+^ mice [62, 192].

Meanwhile, the tight correlation between chemotherapy-induced tissue damage and pyroptosis also provides a promising strategy to reduce toxicity by inhibition of pyroptosis. For instance, DOX, a common antineoplastic agent, can cause cardiotoxicity via pyroptosis in clinical practice [193–195]. MCC950, an NLRP3 inflammasome inhibitor, could obviously suppress myocardial inflammation and fibrosis by inhibiting pyroptosis of cardiomyocyte in DOX-treated mice [193]. Similarly, tripartite motif containing 25 (TRIM25) with E3 ubiquitin ligase activities, could ubiquitinate NLRP1 in DOX-induced cardiomyocyte pyroptosis in vivo, thus protecting against myocardial damage [194]. These studies prompt us that co-treatment of anti-pyroptosis agents, such as MCC950 or TRIM25, may attenuate the myocardial injury induced by chemotherapies in cancer patients.

**Fig. 7:**
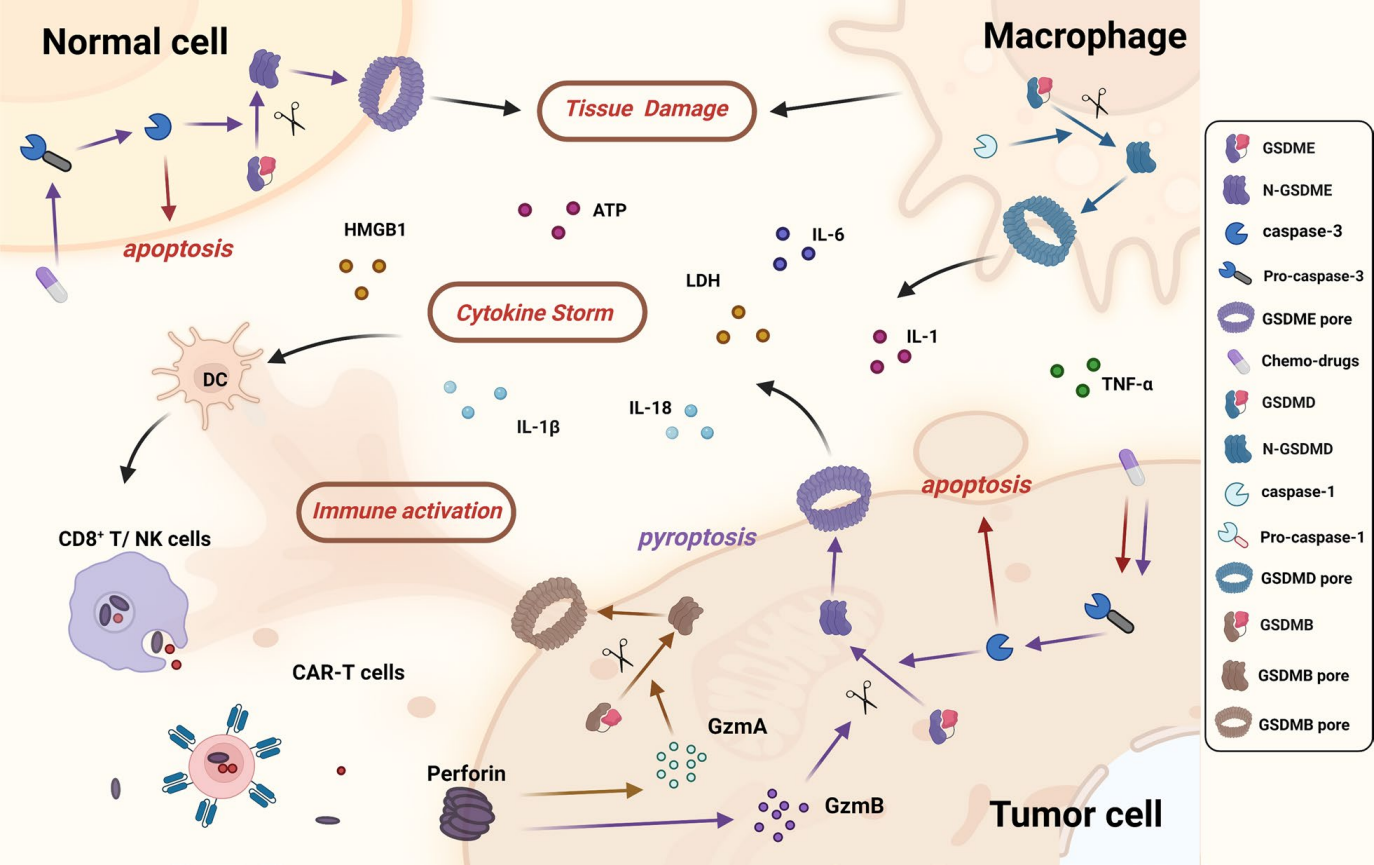
Adverse effects of chemotherapy drugs and nonchemotherapy drugs in pyroptosis pathway. **a** Tissue damage: pyroptosis triggered by chemotherapy drugs in normal cells with high GSDME-expression contributes to their cytotoxicity mainly via GSDME-mediated pyroptosis. **b** Cytokine storm: immune cells like CD8 + T cells and NK cells release a large amount of perforin, GzmA and GzmB, thus promoting pyroptosis and pore-forming process. Cytokines like IL-1β, IL-18, ATP, LDH, and HMGB1 are released into the intercellular substance which subsequently activate immune cells, resulting in severe positive feedback regulatory between immune response and pyroptosis. Activation of pyroptosis in macrophages can release IL-1, IL-6, and TNF-α, thus further exacerbating cytokine storm

CRS, characterized by excessive production of pro-inflammatory cytokines, is another adverse effect of pyroptosis-related therapy [94, 196]. Studies found that immune cells like CD8 + T cells and NK cells release large amount of perforin, GzmA and GzmB which can, respectively, cleave GSDMB and GSDME, thus further promoting pyroptosis and pore-forming process [95, 96]. As a result, cytokines like IL-1β, IL-18, ATP, LDH, and HMGB1 are released into the intercellular substance which subsequently activates immune cells like macrophages, dendritic cells, and NK cells, resulting in severe positive feedback regulatory between immune response and pyroptosis. Activation of pyroptosis in macrophages may release IL-1, IL-6, and TNF-α into serum, thus further exacerbating cytokine storm [197, 198]. One study confirmed CRS occurrence obviously decreased when depleting macrophages, knocking out GSDME, or inhibiting caspase-1 [94], which further affirmed the relationship between immune response and pyroptosis (Fig. 7).

### Challenges and perspectives

Pyroptosis related therapies can undoubtedly affect tumorigenesis, metastasis, proliferation, and invasion [31, 32, 199]. To date, most pyroptosis-related studies are focused on adult malignancies, in contrast to inadequate attention to other common childhood tumors including hepatoblastoma, Wilms tumor, and germ cell tumor. But still, the pharmacological effect of pyroptosis can eliminate malignant tumor cells and has been emerging for treatment of cancers [77, 200]. Cytotoxic therapy is still the mainstream of pyroptosis-related treatments in pediatric malignancies, though with limited efficacy and obvious side effects [77]. Few identified tumor-specific regulatory mechanisms and inconsistent results concerning the role of pyroptosis in tumors may indicate an extra-high heterogeneity and complex microenvironment in pediatric cancers. Before implementation of pyroptosis-related therapies in clinical practice, multiple challenges remain to be addressed.

First, GSDME is hardly expressed in most tumor cells, but abundantly expressed in normal cells [62, 170, 201]. However, as previous studies showed, GSDME is the target of most chemotherapies. The distribution of GSDME may lead to poor efficacy and inevitable cytotoxicity or tissue damage. But gasdermin family proteins are still the potential biomarkers of tumor immunotherapy if we can upregulate gasdermin levels in tumor cells. Decitabine, a DNA methylation inhibitor drug which can upregulate GSDME expression in several cancers and increase their sensitivity to chemotherapy drug-induced pyroptosis, is undoubtedly a promising candidate [171, 172]. Additionally, the exploration of small molecule drugs and the study of specific targeting carriers to target pyroptosis also shed good insights for pediatric cancer treatments.

Second, scant attention has been paid to childhood malignancies in research on pyroptosis-related treatments. Despite some commonalities, pediatric cancers are fundamentally different from their adult counterparts [202]. Originated from embryonal cells, most of pediatric cancers are characterized by low mutational burden and relative lack of neoantigen expression, which wholly restrict their susceptibility to many chemical therapy drugs. This may also be the reason why there are few relevant studies regarding pyroptosis in pediatric cancers.

Third, the immune response to the therapies might also be totally different in children and adults, indicate that the results learned from the latter may not be that applicable to the former. Combination of different therapies may mitigate the challenges. For instance, pembrolizumab and ipilimumab, the common checkpoint inhibitors, can promote the proliferation of cytotoxic T-lymphocyte via programmed cell death protein 1 (PD-1) inhibition and maintain the active state of these cells via cytotoxic T-lymphocyte-associated antigen 4 (CTLA-4) inhibition, respectively [203–205]. The concurrent inhibition of PD-1/CTLA-4 signaling therapy has shown improved anti-tumor efficacy and acceptable safety profile in a phase I adult metastatic melanoma trial [205, 206]. Combination of different pyroptosis-related therapies may also worth exploration, but we should cautiously take the toxicity and safety of the young age into account.

## Conclusions

Accumulating evidences have confirmed an important role of pyroptosis in pediatric cancers. Although it is justifiable that severe side effects and inconsistent mechanism are still challenges that need to be fully elucidated, the future interpretation of pyroptosis in pediatric cancers should be viewed with optimism. Repurposing of existing chemo-drugs, development of novel natural products, synthetic bioactive agents, and promising combination of reagents have brought numerous treatment strategies and novel insights into various pediatric malignancies. With deeper understanding of these diseases and tumor microenvironment, mounting therapies will also flourish continuously and come into development. Herein, we describe the molecular mechanisms of pyroptosis, highlight and discuss the opportunities and challenges for regulating pyroptosis pathways through multiple oncologic treatments in the following malignancies: osteosarcoma, neuroblastoma, leukemia, lymphoma, and brain tumors. It is believed that leverage the strengths of these therapies and overcome the adverse effects will hopefully help us with better understanding of pyroptosis in pediatric cancers and enable better care for the young population.

## Data Availability

The data used to support this study are included within the article.

## Abbreviations

ACD: Accidental cell death
ALL: Acute lymphocytic leukemia
AML: Acute myeloid leukemia
ASC: Apoptosis-related speck-like protein
ATP: Adenosine triphosphate
BAFF: B cell-activating factor
BRAFi: BRAF inhibitors
CARD8: Caspase recruitment domain-containing protein 8
CAR-T: Chimeric antigen receptor-T
cFLIP: Cellular FLICE-inhibitory protein
CNS: Central nervous system
CoQ10: Coenzyme Q10
CPF: Chlorpyrifos
CRS: Cytokine release syndrome
CTLA-4: Cytotoxic T-lymphocyte-associated antigen 4
Cx43: Connexin 43
DAMP: Damage-associated molecular pattern
DOX: Doxorubicin
DPP: Dipeptidyl peptidases
eEF-2K: Eukaryotic elongation factor-2 kinase
ER: Endoplasmic reticulum
FADD: Fas-associated death domain
FDA: Food and Drug Administration
GBM: Glioblastoma multiforme
GG: Galangin
GSDM: Gasdermin
GSDMB: Gasdermin B
GSDMC: Gasdermin C
GSDMD: Gasdermin D
GSDME: Gasdermin E
GSDMD-N: Gasdermin D N-termini
GSDME-N: Gasdermin E N-termini
GSH: Glutathione
GzmA: Granzyme A
GzmB: Granzyme B
HL: Hodgkin lymphoma
HMGB1: High-mobility group protein box 1
HUVECs: Human umbilical vein endothelial cells
ICD: Immunogenic cell death
IL: Interleukin
iNKT: Invariant nature killer T
JNK: C-Jun N-terminal kinase
LDH: Lactic dehydrogenase
LncRNA: Long noncoding RNA
LPS: Lipopolysaccharide
MDS: Myelodysplastic syndromes
MEKi: MEK inhibitors
MLKL: Mixed lineage kinase domain-like
MOMP: Mitochondrial outer membrane permeabilization
NADPH: Nicotinamide adenine dinucleotide phosphate
NF-kB: Nuclear factor kB
NHL: Non-Hodgkin lymphoma
NK: Natural killer cells
NLRC4: NLR family CARD domain-containing protein 4
NLRP3: Nod-like receptor protein 3
NLRs: Nod-like receptors
OGD: Oxygen–glucose deprivation
PAMPs: Pathogen-associated molecular patterns
PCD: Programmed cell death
PD-1: Programmed cell death protein 1
PD-L1: Programmed cell death ligand 1
pHGG: Pediatric high-grade gliomas
PRRs: Pattern recognition receptors
PTX: Paclitaxel
rAAV: Recombinant adeno-associated virus
RIP: Receptor-interacting protein
RIPK: Receptor-interacting serine/threonine kinase
ROS: Reactive oxygen species
tBID: Truncated BID
TCD: Tolerogenic cell death
TFBIP: 2-(4′-Trifluoromethyl)-[1,1′-biphenyl]-4-yl)-1H-imidazo[4,5-f] [1, 10] phenanthroline
TLRs: Toll-like receptors
TMZ: Temozolomide
TNF: Tumor necrosis factor
TRIM25: Tripartite motif containing 25

## References

[1] Galluzzi L, Vitale I, Aaronson SA, Abrams JM, Adam D, Agostinis P (2018). Molecular mechanisms of cell death: recommendations of the Nomenclature Committee on Cell Death 2018. Cell Death Differ.

[2] Zebell SG, Dong X (2015). Cell-cycle regulators and cell death in immunity. Cell Host Microbe.

[3] Deter RL, Baudhuin P, De Duve C (1967). Participation of lysosomes in cellular autophagy induced in rat liver by glucagon. J Cell Biol.

[4] Deter RL, De Duve C (1967). Influence of glucagon, an inducer of cellular autophagy, on some physical properties of rat liver lysosomes. J Cell Biol.

[5] Liu X, Xia S, Zhang Z, Wu H, Lieberman J (2021). Channelling inflammation: gasdermins in physiology and disease. Nat Rev Drug Discov.

[6] Friedlander AM (1986). Macrophages are sensitive to anthrax lethal toxin through an acid-dependent process. J Biol Chem.

[7] Zheng M, Kanneganti TD (2020). The regulation of the ZBP1-NLRP3 inflammasome and its implications in pyroptosis, apoptosis, and necroptosis (PANoptosis). Immunol Rev.

[8] Malireddi RKS, Kesavardhana S, Kanneganti TD (2019). ZBP1 and TAK1: master regulators of NLRP3 inflammasome/pyroptosis, apoptosis, and necroptosis (PAN-optosis). Front Cell Infect Microbiol.

[9] Holler N, Zaru R, Micheau O, Thome M, Attinger A, Valitutti S (2000). Fas triggers an alternative, caspase-8-independent cell death pathway using the kinase RIP as effector molecule. Nat Immunol.

[10] Yang WS, Stockwell BR (2016). Ferroptosis: death by lipid peroxidation. Trends Cell Biol.

[11] Dixon SJ, Lemberg KM, Lamprecht MR, Skouta R, Zaitsev EM, Gleason CE (2012). Ferroptosis: an iron-dependent form of nonapoptotic cell death. Cell.

[12] Kahlson MA, Dixon SJ (2022). Copper-induced cell death. Science.

[13] Tang D, Chen X, Kroemer G (2022). Cuproptosis: a copper-triggered modality of mitochondrial cell death. Cell Res.

[14] David KK, Andrabi SA, Dawson TM, Dawson VL (2009). Parthanatos, a messenger of death. Front Biosci (Landmark Ed).

[15] Delettre C, Yuste VJ, Moubarak RS, Bras M, Lesbordes-Brion JC, Petres S (2006). AIFsh, a novel apoptosis-inducing factor (AIF) pro-apoptotic isoform with potential pathological relevance in human cancer. J Biol Chem.

[16] Liu J, Kuang F, Kang R, Tang D (2020). Alkaliptosis: a new weapon for cancer therapy. Cancer Gene Ther.

[17] Song X, Zhu S, Xie Y, Liu J, Sun L, Zeng D (2018). JTC801 induces pH-dependent death specifically in cancer cells and slows growth of tumors in mice. Gastroenterology.

[18] Shi J, Zhao Y, Wang K, Shi X, Wang Y, Huang H (2015). Cleavage of GSDMD by inflammatory caspases determines pyroptotic cell death. Nature.

[19] Zhao J, Jiang P, Guo S, Schrodi SJ, He D (2021). Apoptosis, autophagy, NETosis, necroptosis, and pyroptosis mediated programmed cell death as targets for innovative therapy in rheumatoid arthritis. Front Immunol.

[20] Frank D, Vince JE (2019). Pyroptosis versus necroptosis: similarities, differences, and crosstalk. Cell Death Differ.

[21] Venkataramany AS, Schieffer KM, Lee K, Cottrell CE, Wang PY, Mardis ER (2022). Alternative RNA splicing defects in pediatric cancers: new insights in tumorigenesis and potential therapeutic vulnerabilities. Ann Oncol.

[22] Gao Y, Volegova M, Nasholm N, Das S, Kwiatkowski N, Abraham BJ (2021). Synergistic anti-tumor effect of combining selective CDK7 and BRD4 inhibition in neuroblastoma. Front Oncol.

[23] Zhang W, He L, Liu Z, Ren X, Qi L, Wan L (2020). Multifaceted functions and novel insight into the regulatory role of RNA N(6)-methyladenosine modification in musculoskeletal disorders. Front Cell Dev Biol.

[24] Cunningham RM, Walton MA, Carter PM (2018). The major causes of death in children and adolescents in the United States. N Engl J Med.

[25] Chen Y, Miao L, Lin H, Zhuo Z, He J (2022). The role of m6A modification in pediatric cancer. Biochim Biophys Acta Rev Cancer.

[26] Force LM, Abdollahpour I, Advani SM, Agius D, Ahmadian E, Alahdab F, Alam T, Alebel A, Alipour V, Allen CA, Almasi-Hashiani A (2019). The global burden of childhood and adolescent cancer in 2017: an analysis of the Global Burden of Disease Study 2017. Lancet Oncol.

[27] Barone A, Casey D, McKee AE, Reaman G (2019). Cancer drugs approved for use in children: impact of legislative initiatives and future opportunities. Pediatr Blood Cancer.

[28] Cerella C, Teiten MH, Radogna F, Dicato M, Diederich M (2014). From nature to bedside: pro-survival and cell death mechanisms as therapeutic targets in cancer treatment. Biotechnol Adv.

[29] Shi J, Gao W, Shao F (2017). Pyroptosis: gasdermin-mediated programmed necrotic cell death. Trends Biochem Sci.

[30] Ren LW, Li W, Zheng XJ, Liu JY, Yang YH, Li S (2021). Benzimidazoles induce concurrent apoptosis and pyroptosis of human glioblastoma cells via arresting cell cycle. Acta Pharmacol Sinica..

[31] Thi HTH, Hong S (2017). Inflammasome as a therapeutic target for cancer prevention and treatment. J Cancer Prev.

[32] Solinas G, Marchesi F, Garlanda C, Mantovani A, Allavena P (2010). Inflammation-mediated promotion of invasion and metastasis. Cancer Metastasis Rev.

[33] Ding Q, Zhang W, Cheng C, Mo F, Chen L, Peng G (2020). Dioscin inhibits the growth of human osteosarcoma by inducing G2/M-phase arrest, apoptosis, and GSDME-dependent cell death in vitro and in vivo. J Cell Physiol.

[34] Lin R, Wei H, Wang S, Huang Z, Chen H, Zhang S (2020). Gasdermin D expression and clinicopathologic outcome in primary osteosarcoma patients. Int J Clin Exp Pathol.

[35] Bu X, Liu J, Ding R, Li Z (2021). Prognostic value of a pyroptosis-related long noncoding RNA signature associated with osteosarcoma microenvironment. J Oncol.

[36] Zhang Y, He R, Lei X, Mao L, Jiang P, Ni C (2021). A novel pyroptosis-related signature for predicting prognosis and indicating immune microenvironment features in osteosarcoma. Front Genet.

[37] Kang X, Jiang L, Chen X, Wang X, Gu S, Wang J (2021). Exosomes derived from hypoxic bone marrow mesenchymal stem cells rescue OGD-induced injury in neural cells by suppressing NLRP3 inflammasome-mediated pyroptosis. Exp Cell Res.

[38] Zhang J, Chen Y, He Q (2020). Distinct characteristics of dasatinib-induced pyroptosis in gasdermin E-expressing human lung cancer A549 cells and neuroblastoma SH-SY5Y cells. Oncol Lett.

[39] Zhao MW, Yang P, Zhao LL (2019). Chlorpyrifos activates cell pyroptosis and increases susceptibility on oxidative stress-induced toxicity by miR-181/SIRT1/PGC-1α/Nrf2 signaling pathway in human neuroblastoma SH-SY5Y cells: Implication for association between chlorpyrifos and Parkinson's disease. Environ Toxicol.

[40] Tang R, Xu J, Zhang B, Liu J, Liang C, Hua J (2020). Ferroptosis, necroptosis, and pyroptosis in anticancer immunity. J Hematol Oncol.

[41] Zheng M, Karki R, Kancharana B, Berns H, Pruett-Miller SM, Kanneganti TD (2021). Caspase-6 promotes activation of the caspase-11-NLRP3 inflammasome during gram-negative bacterial infections. J Biol Chem.

[42] Eisfeld HS, Simonis A, Winter S, Chhen J, Stroh LJ, Krey T (2021). Viral glycoproteins induce NLRP3 inflammasome activation and pyroptosis in macrophages. Viruses.

[43] Xing Y, Zhao J, Zhou M, Jing S, Zhao X, Mao P (2021). The LPS induced pyroptosis exacerbates BMPR2 signaling deficiency to potentiate SLE-PAH. FASEB J.

[44] Gao YL, Zhai JH, Chai YF (2018). Recent advances in the molecular mechanisms underlying pyroptosis in sepsis. Mediat Inflamm.

[45] Hersh D, Monack DM, Smith MR, Ghori N, Falkow S, Zychlinsky A (1999). The Salmonella invasin SipB induces macrophage apoptosis by binding to caspase-1. Proc Natl Acad Sci U S A.

[46] Kijima M, Mizuta R (2019). Histone H1 quantity determines the efficiencies of apoptotic DNA fragmentation and chromatin condensation. Biomed Res.

[47] Xu YJ, Zheng L, Hu YW, Wang Q (2018). Pyroptosis and its relationship to atherosclerosis. Clin Chim Acta.

[48] Walker PR, Leblanc J, Smith B, Pandey S, Sikorska M (1999). Detection of DNA fragmentation and endonucleases in apoptosis. Methods.

[49] Chen X, Zeh HJ, Kang R, Kroemer G, Tang D (2021). Cell death in pancreatic cancer: from pathogenesis to therapy. Nat Rev Gastroenterol Hepatol.

[50] Wang Y, Peng J, Xie X, Zhang Z, Li M, Yang M (2021). Gasdermin E-mediated programmed cell death: an unpaved path to tumor suppression. J Cancer.

[51] Tonnus W, Belavgeni A, Beuschlein F, Eisenhofer G, Fassnacht M, Kroiss M (2021). The role of regulated necrosis in endocrine diseases. Nat Rev Endocrinol.

[52] Gong Y, Fan Z, Luo G, Yang C, Huang Q, Fan K (2019). The role of necroptosis in cancer biology and therapy. Mol Cancer.

[53] Wang Y, Kanneganti TD (2021). From pyroptosis, apoptosis and necroptosis to PANoptosis: a mechanistic compendium of programmed cell death pathways. Comput Struct Biotechnol J.

[54] Jiang W, Deng Z, Dai X, Zhao W (2021). PANoptosis: a new insight into oral infectious diseases. Front Immunol.

[55] Jiang M, Qi L, Li L, Wu Y, Song D, Li Y (2021). Caspase-8: a key protein of cross-talk signal way in "PANoptosis" in cancer. Int J Cancer.

[56] Qiu Y, Cao Y, Cao W, Jia Y, Lu N (2020). The application of ferroptosis in diseases. Pharmacol Res.

[57] Mao C, Liu X, Zhang Y, Lei G, Yan Y, Lee H (2021). DHODH-mediated ferroptosis defence is a targetable vulnerability in cancer. Nature.

[58] Ubellacker JM, Tasdogan A, Ramesh V, Shen B, Mitchell EC, Martin-Sandoval MS (2020). Lymph protects metastasizing melanoma cells from ferroptosis. Nature.

[59] Tsvetkov P, Coy S, Petrova B, Dreishpoon M, Verma A, Abdusamad M (2022). Copper induces cell death by targeting lipoylated TCA cycle proteins. Science.

[60] Lin L, Zhang MX, Zhang L, Zhang D, Li C, Li YL (2021). Autophagy, pyroptosis, and ferroptosis: new regulatory mechanisms for atherosclerosis. Front Cell Dev Biol.

[61] D'Arcy MS (2019). Cell death: a review of the major forms of apoptosis, necrosis and autophagy. Cell Biol Int.

[62] Wang Y, Gao W, Shi X, Ding J, Liu W, He H (2017). Chemotherapy drugs induce pyroptosis through caspase-3 cleavage of a gasdermin. Nature.

[63] Ball B, Zeidan A, Gore SD, Prebet T (2017). Hypomethylating agent combination strategies in myelodysplastic syndromes: hopes and shortcomings. Leuk Lymphoma.

[64] Zhang CC, Li CG, Wang YF, Xu LH, He XH, Zeng QZ (2019). Chemotherapeutic paclitaxel and cisplatin differentially induce pyroptosis in A549 lung cancer cells via caspase-3/GSDME activation. Apoptosis.

[65] Yu J, Li S, Qi J, Chen Z, Wu Y, Guo J (2019). Cleavage of GSDME by caspase-3 determines lobaplatin-induced pyroptosis in colon cancer cells. Cell Death Dis.

[66] Yu P, Wang HY, Tian M, Li AX, Chen XS, Wang XL (2019). Eukaryotic elongation factor-2 kinase regulates the cross-talk between autophagy and pyroptosis in doxorubicin-treated human melanoma cells in vitro. Acta Pharmacol Sin.

[67] Kong Y, Feng Z, Chen A, Qi Q, Han M, Wang S (2019). The natural flavonoid galangin elicits apoptosis, pyroptosis, and autophagy in glioblastoma. Front Oncol.

[68] Yue E, Tuguzbaeva G, Chen X, Qin Y, Li A, Sun X (2019). Anthocyanin is involved in the activation of pyroptosis in oral squamous cell carcinoma. Phytomedicine.

[69] Fan D, Yang Z, Yuan Y, Wu QQ, Xu M, Jin YG (2017). Sesamin prevents apoptosis and inflammation after experimental myocardial infarction by JNK and NF-*κ*B pathways. Food Funct.

[70] Zhang H, Liao X, Wu X, Shi C, Zhang Y, Yuan Y (2022). Iridium(III) complexes entrapped in liposomes trigger mitochondria-mediated apoptosis and GSDME-mediated pyroptosis. J Inorg Biochem.

[71] Jiang Z, Yao L, Ma H, Xu P, Li Z, Guo M (2017). miRNA-214 inhibits cellular proliferation and migration in glioma cells targeting caspase 1 involved in pyroptosis. Oncol Res.

[72] Lu Y, He W, Huang X, He Y, Gou X, Liu X (2021). Strategies to package recombinant Adeno-Associated Virus expressing the N-terminal gasdermin domain for tumor treatment. Nat Commun.

[73] Erkes DA, Cai W, Sanchez IM, Purwin TJ, Rogers C, Field CO (2020). Mutant BRAF and MEK inhibitors regulate the tumor immune microenvironment via pyroptosis. Cancer Discov.

[74] Shi Z, Yuan S, Shi L, Li J, Ning G, Kong X (2021). Programmed cell death in spinal cord injury pathogenesis and therapy. Cell Prolif.

[75] Li Z, Liu W, Fu J, Cheng S, Xu Y, Wang Z (2021). Shigella evades pyroptosis by arginine ADP-riboxanation of caspase-11. Nature.

[76] An S, Hu H, Li Y, Hu Y (2020). Pyroptosis plays a role in osteoarthritis. Aging Dis.

[77] Yu P, Zhang X, Liu N, Tang L, Peng C, Chen X (2021). Pyroptosis: mechanisms and diseases. Signal Transduct Target Ther.

[78] He Y, Amer AO (2014). Microbial modulation of host apoptosis and pyroptosis. Front Cell Infect Microbiol.

[79] Liston A, Masters SL (2017). Homeostasis-altering molecular processes as mechanisms of inflammasome activation. Nat Rev Immunol.

[80] Jing W, Lo Pilato J, Kay C, Man SM (2021). Activation mechanisms of inflammasomes by bacterial toxins. Cell Microbiol.

[81] Bauernfeind FG, Horvath G, Stutz A, Alnemri ES, MacDonald K, Speert D (2009). Cutting edge: NF-kappaB activating pattern recognition and cytokine receptors license NLRP3 inflammasome activation by regulating NLRP3 expression. J Immunol.

[82] Xing Y, Yao X, Li H, Xue G, Guo Q, Yang G (2017). Cutting edge: TRAF6 mediates TLR/IL-1R signaling-induced nontranscriptional priming of the NLRP3 inflammasome. J Immunol.

[83] Orlowski GM, Colbert JD, Sharma S, Bogyo M, Robertson SA, Rock KL (2015). Multiple cathepsins promote pro-IL-1β synthesis and NLRP3-mediated IL-1β activation. J Immunol.

[84] Samways DS, Li Z, Egan TM (2014). Principles and properties of ion flow in P2X receptors. Front Cell Neurosci.

[85] Lee GS, Subramanian N, Kim AI, Aksentijevich I, Goldbach-Mansky R, Sacks DB (2012). The calcium-sensing receptor regulates the NLRP3 inflammasome through Ca2+ and cAMP. Nature.

[86] Schmid-Burgk JL, Gaidt MM, Schmidt T, Ebert TS, Bartok E, Hornung V (2015). Caspase-4 mediates non-canonical activation of the NLRP3 inflammasome in human myeloid cells. Eur J Immunol.

[87] Beckwith KS, Beckwith MS, Ullmann S, Sætra RS, Kim H, Marstad A (2020). Plasma membrane damage causes NLRP3 activation and pyroptosis during *Mycobacterium tuberculosis* infection. Nat Commun.

[88] Baker PJ, Boucher D, Bierschenk D, Tebartz C, Whitney PG, D'Silva DB (2015). NLRP3 inflammasome activation downstream of cytoplasmic LPS recognition by both caspase-4 and caspase-5. Eur J Immunol.

[89] Rühl S, Broz P (2015). Caspase-11 activates a canonical NLRP3 inflammasome by promoting K(+) efflux. Eur J Immunol.

[90] Yang D, He Y, Muñoz-Planillo R, Liu Q, Núñez G (2015). Caspase-11 Requires the pannexin-1 channel and the purinergic P2X7 pore to mediate pyroptosis and endotoxic shock. Immunity.

[91] Orning P, Weng D, Starheim K, Ratner D, Best Z, Lee B (2018). Pathogen blockade of TAK1 triggers caspase-8-dependent cleavage of gasdermin D and cell death. Science.

[92] Hou J, Zhao R, Xia W, Chang CW, You Y, Hsu JM (2020). PD-L1-mediated gasdermin C expression switches apoptosis to pyroptosis in cancer cells and facilitates tumour necrosis. Nat Cell Biol.

[93] Blasco MT, Gomis RR (2020). PD-L1 controls cancer pyroptosis. Nat Cell Biol.

[94] Liu Y, Fang Y, Chen X, Wang Z, Liang X, Zhang T (2020). Gasdermin E-mediated target cell pyroptosis by CAR T cells triggers cytokine release syndrome. Sci Immunol..

[95] Zhang Z, Zhang Y, Xia S, Kong Q, Li S, Liu X (2020). Gasdermin E suppresses tumour growth by activating anti-tumour immunity. Nature.

[96] Zhou Z, He H, Wang K, Shi X, Wang Y, Su Y (2020). Granzyme A from cytotoxic lymphocytes cleaves GSDMB to trigger pyroptosis in target cells. Science.

[97] Wang J, Liu S, Shi J, Li J, Wang S, Liu H (2019). The role of miRNA in the diagnosis, prognosis, and treatment of osteosarcoma. Cancer Biother Radiopharm.

[98] Tu C, Yang K, Wan L, He J, Qi L, Wang W (2020). The crosstalk between lncRNAs and the Hippo signalling pathway in cancer progression. Cell Prolif.

[99] Kansara M, Teng MW, Smyth MJ, Thomas DM (2014). Translational biology of osteosarcoma. Nat Rev Cancer.

[100] Isakoff MS, Bielack SS, Meltzer P, Gorlick R (2015). Osteosarcoma: current treatment and a collaborative pathway to success. J Clin Oncol.

[101] Bhattasali O, Vo AT, Roth M, Geller D, Randall RL, Gorlick R (2015). Variability in the reported management of pulmonary metastases in osteosarcoma. Cancer Med.

[102] Zhang W, Liu Z, Yang Z, Feng C, Zhou X, Tu C (2021). MTHFR polymorphism is associated with severe methotrexate-induced toxicity in osteosarcoma treatment. Front Oncol.

[103] Bai L, Wang S (2014). Targeting apoptosis pathways for new cancer therapeutics. Annu Rev Med.

[104] Zhang C, He J, Qi L, Wan L, Wang W, Tu C (2021). Diagnostic and prognostic significance of dysregulated expression of circular RNAs in osteosarcoma. Expert Rev Mol Diagn.

[105] Misaghi A, Goldin A, Awad M, Kulidjian AA (2018). Osteosarcoma: a comprehensive review. Sicot j.

[106] Franceschini N, Lam SW, Cleton-Jansen AM, Bovée J (2020). What's new in bone forming tumours of the skeleton?. Virchows Arch.

[107] Luetke A, Meyers PA, Lewis I, Juergens H (2014). Osteosarcoma treatment—Where do we stand? A state of the art review. Cancer Treat Rev.

[108] Tu C, He J, Qi L, Ren X, Zhang C, Duan Z (2020). Emerging landscape of circular RNAs as biomarkers and pivotal regulators in osteosarcoma. J Cell Physiol.

[109] Gao J, Qiu X, Xi G, Liu H, Zhang F, Lv T (2018). Downregulation of GSDMD attenuates tumor proliferation via the intrinsic mitochondrial apoptotic pathway and inhibition of EGFR/Akt signaling and predicts a good prognosis in non-small cell lung cancer. Oncol Rep.

[110] Peng J, Jiang H, Guo J, Huang J, Yuan Q, Xie J (2020). CD147 Expression is associated with tumor proliferation in bladder cancer via GSDMD. Biomed Res Int.

[111] Yan H, Luo B, Wu X, Guan F, Yu X, Zhao L (2021). Cisplatin induces pyroptosis via activation of MEG3/NLRP3/caspase-1/GSDMD pathway in triple-negative breast cancer. Int J Biol Sci.

[112] Tsubota S, Kadomatsu K (2018). Origin and initiation mechanisms of neuroblastoma. Cell Tissue Res.

[113] Swift CC, Eklund MJ, Kraveka JM, Alazraki AL (2018). Updates in diagnosis, management, and treatment of neuroblastoma. Radiographics.

[114] Whittle SB, Smith V, Doherty E, Zhao S, McCarty S, Zage PE (2017). Overview and recent advances in the treatment of neuroblastoma. Expert Rev Anticancer Ther.

[115] Pfluger T, Piccardo A (2017). Neuroblastoma: MIBG imaging and new tracers. Semin Nucl Med.

[116] Gains JE, Bomanji JB, Fersht NL, Sullivan T, D'Souza D, Sullivan KP (2011). 177Lu-DOTATATE molecular radiotherapy for childhood neuroblastoma. J Nucl Med.

[117] Eleveld TF, Oldridge DA, Bernard V, Koster J, Colmet Daage L, Diskin SJ (2015). Relapsed neuroblastomas show frequent RAS-MAPK pathway mutations. Nat Genet.

[118] Masuda Y, Futamura M, Kamino H, Nakamura Y, Kitamura N, Ohnishi S (2006). The potential role of DFNA5, a hearing impairment gene, in p53-mediated cellular response to DNA damage. J Hum Genet.

[119] El-Deiry WS (2003). The role of p53 in chemosensitivity and radiosensitivity. Oncogene.

[120] Chen L, Weng B, Li H, Wang H, Li Q, Wei X (2019). A thiopyran derivative with low murine toxicity with therapeutic potential on lung cancer acting through a NF-*κ*B mediated apoptosis-to-pyroptosis switch. Apoptosis.

[121] Feng J, Li M, Wei Q, Li S, Song S, Hua Z (2018). Unconjugated bilirubin induces pyroptosis in cultured rat cortical astrocytes. J Neuroinflammation.

[122] An Q, Fan CH, Xu SM (2017). Recent perspectives of pediatric leukemia: an update. Eur Rev Med Pharmacol Sci.

[123] González-Herrero I, Rodríguez-Hernández G, Luengas-Martínez A, Isidro-Hernández M, Jiménez R, García-Cenador MB (2018). The making of leukemia. Int J Mol Sci.

[124] Metayer C, Milne E, Clavel J, Infante-Rivard C, Petridou E, Taylor M (2013). The childhood leukemia international consortium. Cancer Epidemiol.

[125] Amitay EL, Keinan-Boker L (2015). Breastfeeding and childhood leukemia incidence: a meta-analysis and systematic review. JAMA Pediatr.

[126] Razmara M, Srinivasula SM, Wang L, Poyet JL, Geddes BJ, DiStefano PS (2002). CARD-8 protein, a new CARD family member that regulates caspase-1 activation and apoptosis. J Biol Chem.

[127] Johnson DC, Taabazuing CY, Okondo MC, Chui AJ, Rao SD, Brown FC (2018). DPP8/DPP9 inhibitor-induced pyroptosis for treatment of acute myeloid leukemia. Nat Med.

[128] Linder A, Bauernfried S, Cheng Y, Albanese M, Jung C, Keppler OT (2020). CARD8 inflammasome activation triggers pyroptosis in human T cells. Embo j.

[129] Okondo MC, Johnson DC, Sridharan R, Go EB, Chui AJ, Wang MS (2017). DPP8 and DPP9 inhibition induces pro-caspase-1-dependent monocyte and macrophage pyroptosis. Nat Chem Biol.

[130] Murakami T, Ockinger J, Yu J, Byles V, McColl A, Hofer AM (2012). Critical role for calcium mobilization in activation of the NLRP3 inflammasome. Proc Natl Acad Sci U S A.

[131] Horng T (2014). Calcium signaling and mitochondrial destabilization in the triggering of the NLRP3 inflammasome. Trends Immunol.

[132] Suzuki-Kakisaka H, Sugimoto J, Tetarbe M, Romani AM, Ramirez Kitchen CM, Bernstein HB (2013). Magnesium sulfate increases intracellular magnesium reducing inflammatory cytokine release in neonates. Am J Reprod Immunol.

[133] Chang YY, Kao MC, Lin JA, Chen TY, Cheng CF, Wong CS (2018). Effects of MgSO(4) on inhibiting Nod-like receptor protein 3 inflammasome involve decreasing intracellular calcium. J Surg Res.

[134] Schröder NW, Eckert J, Stübs G, Schumann RR (2008). Immune responses induced by spirochetal outer membrane lipoproteins and glycolipids. Immunobiology.

[135] Luo X, Zhang X, Gan L, Zhou C, Zhao T, Zeng T (2018). The outer membrane protein Tp92 of treponema pallidum induces human mononuclear cell death and IL-8 secretion. J Cell Mol Med.

[136] Neelapu SS, Locke FL, Bartlett NL, Lekakis LJ, Miklos DB, Jacobson CA (2017). Axicabtagene ciloleucel CAR T-Cell therapy in refractory large B-Cell lymphoma. N Engl J Med.

[137] Sadelain M, Rivière I, Riddell S (2017). Therapeutic T cell engineering. Nature.

[138] Wang C, Liu J, Liu Y (2021). Progress in the treatment of HIV-associated lymphoma when combined with the antiretroviral therapies. Front Oncol.

[139] Allen CE, Kelly KM, Bollard CM (2015). Pediatric lymphomas and histiocytic disorders of childhood. Pediatr Clin North Am.

[140] Burkhardt B, Zimmermann M, Oschlies I, Niggli F, Mann G, Parwaresch R (2005). The impact of age and gender on biology, clinical features and treatment outcome of non-Hodgkin lymphoma in childhood and adolescence. Br J Haematol.

[141] Swerdlow SH, Campo E, Pileri SA, Harris NL, Stein H, Siebert R (2016). The 2016 revision of the World Health Organization classification of lymphoid neoplasms. Blood.

[142] Qiang L, Yuan J, Shouyin J, Yulin L, Libing J, Jian-An W (2016). Sesamin attenuates lipopolysaccharide-induced acute lung injury by inhibition of TLR4 signaling pathways. Inflammation.

[143] Xu P, Cai F, Liu X, Guo L (2015). Sesamin inhibits lipopolysaccharide-induced proliferation and invasion through the p38-MAPK and NF-*κ*B signaling pathways in prostate cancer cells. Oncol Rep.

[144] Meng Z, Liu H, Zhang J, Zheng Z, Wang Z, Zhang L (2021). Sesamin promotes apoptosis and pyroptosis via autophagy to enhance antitumour effects on murine T-cell lymphoma. J Pharmacol Sci.

[145] Mackay F, Schneider P (2009). Cracking the BAFF code. Nat Rev Immunol.

[146] Karin M, Delhase M (2000). The I kappa B kinase (IKK) and NF-kappa B: key elements of proinflammatory signalling. Semin Immunol.

[147] Lim KH, Chen LC, Hsu K, Chang CC, Chang CY, Kao CW (2020). BAFF-driven NLRP3 inflammasome activation in B cells. Cell Death Dis.

[148] Lan P, Fan Y, Zhao Y, Lou X, Monsour HP, Zhang X (2017). TNF superfamily receptor OX40 triggers invariant NKT cell pyroptosis and liver injury. J Clin Invest.

[149] Udaka YT, Packer RJ (2018). Pediatric brain tumors. Neurol Clin.

[150] Jones C, Karajannis MA, Jones DTW, Kieran MW, Monje M, Baker SJ (2017). Pediatric high-grade glioma: biologically and clinically in need of new thinking. Neuro Oncol.

[151] Omuro A, DeAngelis LM (2013). Glioblastoma and other malignant gliomas: a clinical review. JAMA.

[152] Booth TC, Wiegers EC, Warnert EAH, Schmainda KM, Riemer F, Nechifor RE (2021). High-grade glioma treatment response monitoring biomarkers: a position statement on the evidence supporting the use of advanced MRI techniques in the clinic, and the latest bench-to-bedside developments. Part 2: spectroscopy, chemical exchange saturation, multiparametric imaging, and radiomics. Front Oncol.

[153] Perry JR, Laperriere N, O'Callaghan CJ, Brandes AA, Menten J, Phillips C (2017). Short-course radiation plus temozolomide in elderly patients with glioblastoma. N Engl J Med.

[154] Karachi A, Dastmalchi F, Mitchell DA, Rahman M (2018). Temozolomide for immunomodulation in the treatment of glioblastoma. Neuro Oncol.

[155] Lu TX, Rothenberg ME (2018). MicroRNA. J Allergy Clin Immunol.

[156] Brennecke J, Hipfner DR, Stark A, Russell RB, Cohen SM (2003). Bantam encodes a developmentally regulated microRNA that controls cell proliferation and regulates the proapoptotic gene hid in drosophila. Cell.

[157] Saliminejad K, Khorram Khorshid HR, Soleymani Fard S, Ghaffari SH (2019). An overview of microRNAs: biology, functions, therapeutics, and analysis methods. J Cell Physiol.

[158] Tang SL, Gao YL, Chen XB (2015). MicroRNA-214 targets PCBP2 to suppress the proliferation and growth of glioma cells. Int J Clin Exp Pathol.

[159] Wang S, Jiao B, Geng S, Ma S, Liang Z, Lu S (2014). Combined aberrant expression of microRNA-214 and UBC9 is an independent unfavorable prognostic factor for patients with gliomas. Med Oncol.

[160] Carneiro BA, El-Deiry WS (2020). Targeting apoptosis in cancer therapy. Nat Rev Clin Oncol.

[161] Hanahan D, Weinberg RA (2011). Hallmarks of cancer: the next generation. Cell.

[162] Lee SY, Ju MK, Jeon HM, Jeong EK, Lee YJ, Kim CH (2018). Regulation of tumor progression by programmed necrosis. Oxid Med Cell Longev.

[163] Chen X, Kang R, Kroemer G, Tang D (2021). Broadening horizons: the role of ferroptosis in cancer. Nat Rev Clin Oncol.

[164] Fu L, Jin W, Zhang J, Zhu L, Lu J, Zhen Y (2022). Repurposing non-oncology small-molecule drugs to improve cancer therapy: current situation and future directions. Acta Pharm Sin B.

[165] Versteijne E, de Hingh I, Homs MYV, Intven MPW, Klaase JM, van Santvoort HC (2021). Neoadjuvant treatment for resectable and borderline resectable pancreatic cancer: Chemotherapy or chemoradiotherapy?. Front Oncol.

[166] Rogers C, Fernandes-Alnemri T, Mayes L, Alnemri D, Cingolani G, Alnemri ES (2017). Cleavage of DFNA5 by caspase-3 during apoptosis mediates progression to secondary necrotic/pyroptotic cell death. Nat Commun.

[167] Kim MS, Chang X, Yamashita K, Nagpal JK, Baek JH, Wu G (2008). Aberrant promoter methylation and tumor suppressive activity of the DFNA5 gene in colorectal carcinoma. Oncogene.

[168] Yokomizo K, Harada Y, Kijima K, Shinmura K, Sakata M, Sakuraba K (2012). Methylation of the DFNA5 gene is frequently detected in colorectal cancer. Anticancer Res.

[169] Wang CJ, Tang L, Shen DW, Wang C, Yuan QY, Gao W (2013). The expression and regulation of DFNA5 in human hepatocellular carcinoma DFNA5 in hepatocellular carcinoma. Mol Biol Rep.

[170] Akino K, Toyota M, Suzuki H, Imai T, Maruyama R, Kusano M (2007). Identification of DFNA5 as a target of epigenetic inactivation in gastric cancer. Cancer Sci.

[171] Diesch J, Le Pannerer MM, Winkler R, Casquero R, Muhar M, van der Garde M (2021). Inhibition of CBP synergizes with the RNA-dependent mechanisms of azacitidine by limiting protein synthesis. Nat Commun.

[172] Ma J, Ge Z (2021). Comparison between decitabine and azacitidine for patients with acute myeloid leukemia and higher-risk myelodysplastic syndrome: a systematic review and network meta-analysis. Front Pharmacol.

[173] Chafouleas JG, Pardue RL, Brinkley BR, Dedman JR, Means AR (1981). Regulation of intracellular levels of calmodulin and tubulin in normal and transformed cells. Proc Natl Acad Sci U S A.

[174] Ashour AA, Abdel-Aziz AA, Mansour AM, Alpay SN, Huo L, Ozpolat B (2014). Targeting elongation factor-2 kinase (eEF-2K) induces apoptosis in human pancreatic cancer cells. Apoptosis.

[175] Molina-Crespo Á, Cadete A, Sarrio D, Gámez-Chiachio M, Martinez L, Chao K (2019). Intracellular delivery of an antibody targeting gasdermin-B reduces HER2 breast cancer aggressiveness. Clin Cancer Res.

[176] Schauder DM, Kim J, Nijhawan RI (2020). Evaluation of the use of capecitabine for the treatment and prevention of actinic keratoses, squamous cell carcinoma, and basal cell carcinoma: a systematic review. JAMA Dermatol.

[177] Li M, Tang D, Yang T, Qian D, Xu R (2021). Apoptosis triggering, an important way for natural products from herbal medicines to treat pancreatic cancers. Front Pharmacol.

[178] Tilaoui M, Ait Mouse H, Zyad A (2021). Update and New Insights on Future Cancer Drug Candidates From Plant-Based Alkaloids. Front Pharmacol.

[179] Kosakowska-Cholody T, Lin J, Srideshikan SM, Scheffer L, Tarasova NI, Acharya JK (2014). HKH40A downregulates GRP78/BiP expression in cancer cells. Cell Death Dis.

[180] Crittenden MR, Zebertavage L, Kramer G, Bambina S, Friedman D, Troesch V (2018). Tumor cure by radiation therapy and checkpoint inhibitors depends on pre-existing immunity. Sci Rep.

[181] Zhao X, Shao C. Radiotherapy-mediated immunomodulation and anti-tumor abscopal effect combining immune checkpoint blockade. Cancers (Basel). 2020;12(10). 10.3390/cancers12102762 PMC7600068 32992835

[182] Golden EB, Frances D, Pellicciotta I, Demaria S, Helen Barcellos-Hoff M, Formenti SC (2014). Radiation fosters dose-dependent and chemotherapy-induced immunogenic cell death. Oncoimmunology.

[183] Vasudevan HN, Yom SS (2020). Combining systemic therapy with radiation: head and neck cancer treatments in an era of targeted agents and immunotherapy. J Natl Compr Canc Netw.

[184] Herrera FG, Irving M, Kandalaft LE, Coukos G (2019). Rational combinations of immunotherapy with radiotherapy in ovarian cancer. Lancet Oncol.

[185] Ko EC, Raben D, Formenti SC (2018). The integration of radiotherapy with immunotherapy for the treatment of non-small cell lung cancer. Clin Cancer Res.

[186] Hu B, Jin C, Li HB, Tong J, Ouyang X, Cetinbas NM (2016). The DNA-sensing AIM2 inflammasome controls radiation-induced cell death and tissue injury. Science.

[187] Li C, Tian M, Gou Q, Jia YR, Su X (2019). Connexin43 modulates X-ray-induced pyroptosis in human umbilical vein endothelial cells. Biomed Environ Sci.

[188] Wu D, Han R, Deng S, Liu T, Zhang T, Xie H (2018). Protective effects of flagellin A N/C against radiation-induced NLR pyrin domain containing 3 inflammasome-dependent pyroptosis in intestinal cells. Int J Radiat Oncol Biol Phys.

[189] Liu YG, Chen JK, Zhang ZT, Ma XJ, Chen YC, Du XM (2017). NLRP3 inflammasome activation mediates radiation-induced pyroptosis in bone marrow-derived macrophages. Cell Death Dis.

[190] Liu T, Wu DM, Zhang F, Zhang T, He M, Zhao YY (2022). miR-142a-3p enhances FlaA N/C protection against radiation-mediated intestinal injury by modulating the IRAK1/NF-kappaB signaling pathway. Int J Radiat Oncol Biol Phys.

[191] Xiao Y, Zhang T, Ma X, Yang QC, Yang LL, Yang SC (2021). Microenvironment-responsive prodrug-induced pyroptosis boosts cancer immunotherapy. Adv Sci (Weinh).

[192] Yu X, He S (2017). GSDME as an executioner of chemotherapy-induced cell death. Sci China Life Sci.

[193] Zhang L, Jiang YH, Fan C, Zhang Q, Jiang YH, Li Y (2021). MCC950 attenuates doxorubicin-induced myocardial injury in vivo and in vitro by inhibiting NLRP3-mediated pyroptosis. Biomed Pharmacother.

[194] Wang X, Lian Z, Ge Y, Yu D, Li S, Tan K (2021). TRIM25 rescues against doxorubicin-induced pyroptosis through promoting NLRP1 ubiquitination. Cardiovasc Toxicol.

[195] Zhang JM, Yu RQ, Wu FZ, Qiao L, Wu XR, Fu YJ (2021). BMP-2 alleviates heart failure with type 2 diabetes mellitus and doxorubicin-induced AC16 cell injury by inhibiting NLRP3 inflammasome-mediated pyroptosis. Exp Ther Med.

[196] Zhang J, Wu H, Yao X, Zhang D, Zhou Y, Fu B (2021). Pyroptotic macrophages stimulate the SARS-CoV-2-associated cytokine storm. Cell Mol Immunol.

[197] Wang Q, Wang Y, Ding J, Wang C, Zhou X, Gao W (2020). A bioorthogonal system reveals antitumour immune function of pyroptosis. Nature.

[198] Zhang Z, Zhang Y, Lieberman J (2021). Lighting a fire: can we harness pyroptosis to ignite antitumor immunity?. Cancer Immunol Res.

[199] Fang Y, Tian S, Pan Y, Li W, Wang Q, Tang Y (2020). Pyroptosis: a new frontier in cancer. Biomed Pharmacother.

[200] Tan Y, Chen Q, Li X, Zeng Z, Xiong W, Li G (2021). Pyroptosis: a new paradigm of cell death for fighting against cancer. J Exp Clin Cancer Res.

[201] Van Laer L, Huizing EH, Verstreken M, van Zuijlen D, Wauters JG, Bossuyt PJ (1998). Nonsyndromic hearing impairment is associated with a mutation in DFNA5. Nat Genet.

[202] Meyran D, Terry RL, Zhu JJ, Haber M, Ziegler DS, Ekert PG (2021). Early-phenotype CAR-T cells for the treatment of pediatric cancers. Ann Oncol.

[203] Cacciotti C, Choi J, Alexandrescu S, Zimmerman MA, Cooney TM, Chordas C (2020). Immune checkpoint inhibition for pediatric patients with recurrent/refractory CNS tumors: a single institution experience. J Neurooncol.

[204] MarjaŃska A, Drogosiewicz M, Dembowska-BagiŃska B, PawiŃska-WĄsikowska K, Balwierz W, Bobeff K (2020). Nivolumab for the treatment of advanced pediatric malignancies. Anticancer Res.

[205] Wolchok JD, Kluger H, Callahan MK, Postow MA, Rizvi NA, Lesokhin AM (2013). Nivolumab plus ipilimumab in advanced melanoma. N Engl J Med.

[206] Kyi C, Postow MA (2016). Immune checkpoint inhibitor combinations in solid tumors: opportunities and challenges. Immunotherapy.

